# A study of the role of GATA4 polymorphism in cardiovascular metabolic disorders

**DOI:** 10.1186/1479-7364-7-25

**Published:** 2013-12-12

**Authors:** Nzioka P Muiya, Salma M Wakil, Asma I Tahir, Samya Hagos, Mohammed Najai, Daisy Gueco, Nada Al-Tassan, Editha Andres, Nejat Mazher, Brian F Meyer, Nduna Dzimiri

**Affiliations:** 1 Genetics Department, King Faisal Specialist Hospital and Research Centre, Riyadh 11211, Saudi Arabia

**Keywords:** GATA4 gene polymorphism, Haplotypes, Coronary artery disease, Dyslipidaemia, High-density lipoprotein-cholesterol, Hypercholesterolaemia, Hypertriglyceridaemia, Low-density lipoprotein-cholesterol

## Abstract

**Background:**

The study was designed to evaluate the association of *GATA4* gene polymorphism with coronary artery disease (CAD) and its metabolic risk factors, including dyslipidaemic disorders, obesity, type 2 diabetes and hypertension, following a preliminary study linking early onset of CAD in heterozygous familial hypercholesterolaemia to chromosome 8, which harbours the *GATA4* gene.

**Results:**

We first sequenced the whole *GATA4* gene in 250 individuals to identify variants of interest and then investigated the association of 12 single-nucleotide polymorphisms (SNPs) with the disease traits using Taqman chemistry in 4,278 angiographed Saudi individuals. Of the studied SNPs, rs804280 (1.14 (1.03 to 1.27); *p* = 0.009) was associated with CAD (2,274 cases vs 2,004 controls), hypercholesterolaemia (1,590 vs 2,487) (1.61 (1.03–2.52); *p* = 0.037) and elevated low-density lipoprotein-cholesterol (hLDLC) (575 vs 3,404) (1.87 (1.10–3.15); *p* = 0.020). Additionally, rs3729855_T (1.52 (1.09–2.11; *p* = 0.013)) and rs17153743 (AG + GG) (2.30 (1.30–4.26); *p* = 0.005) were implicated in hypertension (3,312 vs 966), following adjustments for confounders. Furthermore, haplotypes CCCGTGCC (*χ*^2^ = 4.71; *p* = 0.041) and GACCCGTG (*χ*^2^ = 3.84; *p* = 0.050) constructed from the SNPs were associated with CAD and ACCCACGC (*χ*^2^ = 6.58; *p* = 0.010) with myocardial infarction, while hypercholesterolaemia (*χ*^2^ = 3.86; *p* = 0.050) and hLDLC (*χ*^2^ = 4.94; *p* = 0.026) shared the AACCCATGT, and AACCCATGTC was associated with hLDLC (*χ*^2^ = 4.83; *p* = 0.028). A 10-mer GACCCGCGCC (*χ*^2^ = 7.59; *p* = 0.006) was associated with obesity (1,631 vs 2,362), and the GACACACCC (*χ*^2^ = 4.05; *p* = 0.044) was implicated in type 2 diabetes mellitus 2,378 vs 1,900).

**Conclusion:**

Our study implicates GATA4 in CAD and its metabolic risk traits. The finding also points to the possible involvement of yet undefined entities related to GATA4 transcription activity or gene regulatory pathways in events leading to these cardiovascular disorders.

## Background

The GATA binding proteins constitute a family of cell-restricted zinc-finger transcription factors (TFs), which recognize the GATA motif present in the promoters of many genes. This family, comprising six developmental/cell-type specific transcription factors, GATA1-6, is critical to the development of diverse tissues [1-4] and acts in cooperation with more widely expressed factors to direct lineage-specific gene expression [5-11]. Their transcriptional activity is modulated through interactions with nuclear proteins, including the zinc finger proteins of the Kruppel and FOG/U-shaped families, general co-activators of the p300 and cAMP-response element-binding (CREB) protein (CBP), the myocardial-expressed protein Nkx2.5, and NF-AT3 [12-15]. In particular, in the cardiac tissue where its expression is more than 20-fold greater than in other tissues [16], it is a critical regulator of cardiac angiogenesis and gene expression, and is involved in modulating cardiomyocyte differentiation and adaptive responses of the adult heart [5,17,18]. Additionally, GATA4 is abundantly expressed in the endocardium and endothelial cells, pointing to an important regulatory control in their development and function [11,19]. The abundance and localization of the GATA4 to the myocardial tissue suggests a pivotal cardiovascular functional input, which if perturbed, may lead to various cardiac disorders and coronary artery disease (CAD)-related events. However, while the importance of this gene is now well appreciated in congenital heart diseases [20-26] and some other cardiac malformations [27-29], little is known about its potential role in vascular activity-related diseases, such as dyslipidaemia and diabetes mellitus. Dyslipidaemia can be triggered as a result of a genetic predisposition, secondary causes, or a combination of both. However, the genetic causes of this disorder remain to be identified. In a preliminary study investigating the genomic linkage to early onset of CAD in two Saudi families with heterozygous familial hypercholesterolaemia (HFH), we identified a locus on chromosome 8, which harbours the *GATA4* gene, as a plausible candidate for CAD, HFH and harbouring of low high-density lipoprotein levels. This led to the notion that GATA4 presents a potential candidate for CAD onset, especially in dyslipidaemic conditions. Given the lack of information on the role of this gene in the etiology of atherosclerosis, our study sought to comprehensively investigate the likelihood of GATA4 polymorphism predisposing individuals to acquiring the cardiovascular metabolic risk traits, particularly dyslipidaemia, obesity, type 2 diabetes mellitus and hypertension, as a potential trigger for the disease onset related to these disorders. To this effect, we first sequenced the gene in the two HFH families and an additional 250 individuals from the Saudi general population to identify potentially informative variants, and then performed a population-based association study for selected variants with CAD and its risk traits in a larger cohort of angiographed Saudi individuals.

## Results

### Linkage analysis for dyslipidaemia and early onset coronary artery disease

**Figure 1:**
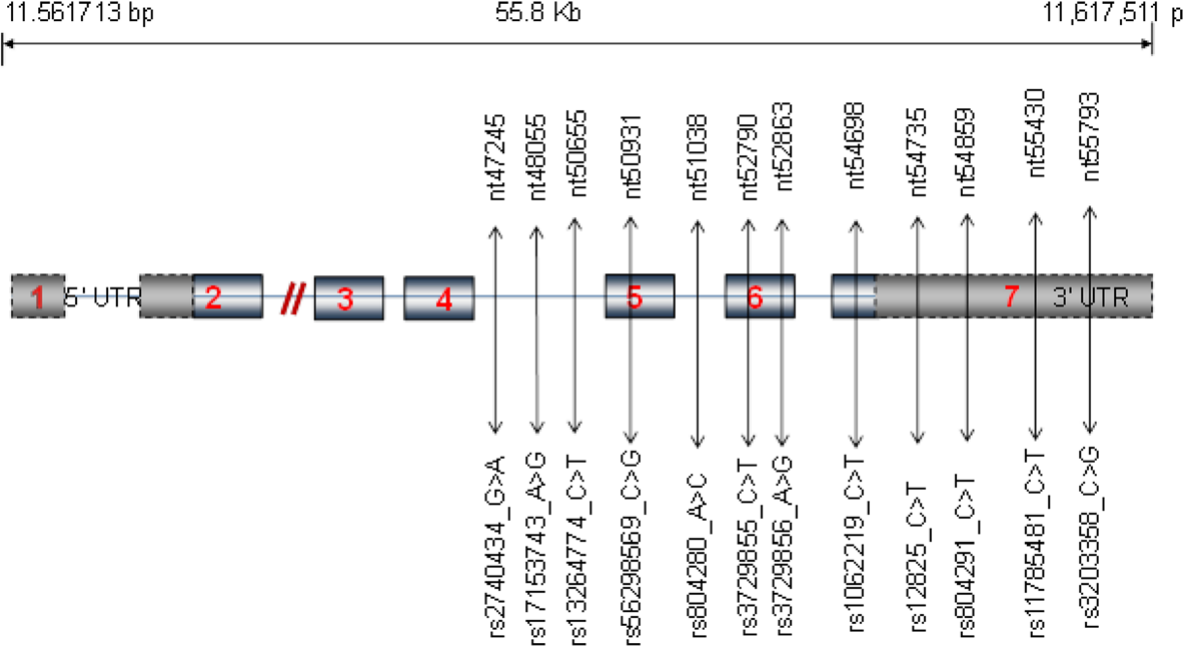
**Schematic of *GATA4 *gene sequence on chromosome 8p23.1.** Diagram shows relative loci of studied variants and their positions (drawn not to scale). Numbers in rectangles represent the exons.

**Figure 2:**
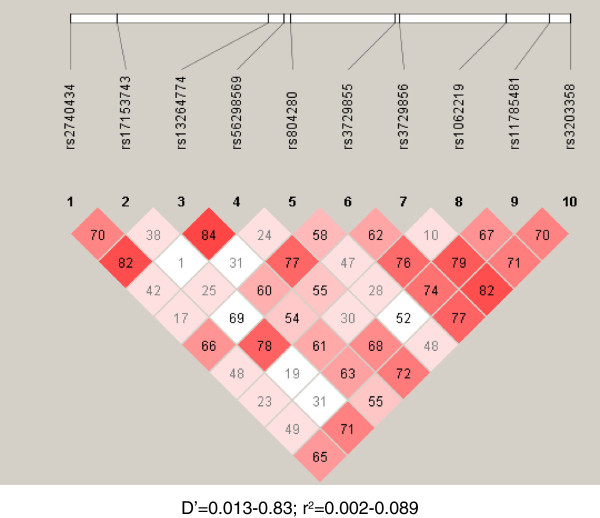
**Linkage disequilibrium (LD) structure of ten SNPs included in haplotyping.** D′, coefficient of linkage disequilibrium; r, regression coefficient.

The initial scanning of the HFH yielded several peaks in different genomic regions including Chromosome (Chr) 8, among others, giving a logarithm of the odds (LOD) score of 1.8 that isolated at least three of the affected siblings with the early onset of CAD in both families. One of the potential culprits at this locus is GATA4, which we elected to pursue further for its role in dyslipidaemia-related onset of CAD. Subsequent sequencing of the genes in all members of the two HFH families and an additional 250 individuals led to the selection of 12 single-nucleotide polymorphisms (SNPs), rs2740434_G > A (minor allele frequency = 0.26), rs17153743_A > G (0.02), rs13264774_C > T (0.14), rs56298569_G > C (0.44), rs804280 A > C (0.04), rs3729855 C > T (0.18), rs3729856_A > G (0.45), rs1062219 C > T (0.45), rs12825_C > G (0.09), rs804291_C > T (0.32), rs11785481 C > T (0.12), rs3203358 C > G (0.20) for further case-control studies. Selection of the SNPs was based partly on the prevalence in our general population and partly on currently available information of their role in the disease. Furthermore, these SNPs reside in the later portion of the gene (also encompassing its three prime untranslated region, 3′-UTR), which encodes the C-terminal of the protein and harbours gene regulatory motifs, both of which are thought to be important in the transcriptional activity of the GATA4 (Figure 1). We were also curious to understand the potential role of changes in the 3′-UTR in the disease. Figure 2 displays the linkage disequilibrium (LD) structure of the ten SNPs included in the haplotyping.

### GATA4 genotyping and disease

Since *GATA4* constitutes an established risk gene for congenital heart disease, it was necessary to rule out the likelihood of such potential relationships masking those pertaining to disease trait-gene interactions under investigation. We therefore first tested for associations of the gene variants with congenital heart disease as an independent patient group (*n* = 113) in our study population, using a homogenous set of individuals with dilated cardiomyopathy (*n* = 272) as controls. The results pointed to a significant causative association for the rs3729856 A > G (p.S377G) (Odds ratio (95% confidence interval) = 1.76 (1.19–2.61); *p* = 0.005), the rs3729856_GG (5.09 (2.14–12.06); *p* < 0.000001), and rs11785481_TT (6.23 (1.93–20.17); *p* < 0.002) with congenital heart disease. Interestingly, in the multivariate analyses for congenital heart disease versus the rest of the studied population (113 cases vs 3,969 non-disease individuals), only the rs11785481_T (1.78 (1.06–3.00); *p* = 0.030) retained its significant association (Table 1), pointing to a strong link that could potentially mask the relationships for the other diseases in our current study setting. Furthermore, multivariate analyses showed an association of rs804280_C (1.14 (1.03–1.27); *p* = 0.009) with CAD (Table 1), while the rs1062219C > T (*p* = 0.036) lost this property, following the adjustment for confounders. Interestingly, the rs804291_TT was associated with both hypercholesterolaemia (hChol) (1.61 (1.03–2.52) and elevated low-density lipoprotein-cholesterol (hLDLC) (1.87 (1.10–3.15); *p* = 0.020). In contrast, the rs3203358_G was protective against acquiring both CAD (0.88 (0.78–0.99); *p* = 0.042) and hChol (0.86 (0.75–0.98); *p* = 0.026) (Table 1). Besides, the rs3729855_T (p.N352N) (1.52 (1.09–2.11) and rs17153743 (AG + GG) (2.30 (1.30–4.26); *p* = 0.005) were implicated in hypertension, while the former appeared to be protective against type 2 diabetes mellitus (0.68 (0.53–0.88); *p* = 0.003) (Table 2), also following the Bonferroni corrections for age, sex, and other confounders (Additional file 1, GATA4 Suppl data).

**Table 1:** Association of GATA4 gene variants with coronary artery disease, myocardial infarction congenital heart disease

**Block**	**Haplotype**	**Pooled**	**Cases**	**Control**	***χ*** **2**	***p*** **value**
Hypercholesterolaemia						
1–9	AACCCATGT	0.063	0.071	0.060	3.86	0.050
5–10	CATGTC	0.088	0.096	0.082	4.25	0.039
High low-density lipoprotein						
1–10	AACCCATGTC	0.062	0.079	0.062	4.83	0.028
	GACCCATGCC	0.015	0.024	0.015	4.94	0.026
1–9	AACCCATGT	0.063	0.078	0.062	4.31	0.038
Hypertriglyceridaemia						
2–7	ACACAT	0.083	0.072	0.088	4.79	0.029
2–6	ACCTA	0.013	0.018	0.011	4.65	0.031
3–6	CCTA	0.016	0.021	0.014	4.34	0.037
1–5	AACCT	0.01	0.015	0.009	4.49	0.034
Hypertension						
1–8	GACCCACG	0.02	0.018	0.026	4.26	0.039
1–6	GACCCA	0.103	0.098	0.119	6.78	0.009
Type 2 diabetes mellitus						
1–9	GACACACCC	0.18	0.192	0.175	4.05	0.044
Obesity						
1–10	GACCCGCGCC	0.025	0.032	0.022	7.59	0.006**
	GACCCGCGC	0.026	0.033	0.022	7.42	0.006**
2–10	ACCCGCGCC	0.026	0.031	0.022	6.55	0.011
3–10	CCCGCGCC	0.026	0.031	0.022	6.55	0.011
1–8	GACCCGCG	0.028	0.033	0.024	5.27	0.022
1–7	GACCCGC	0.028	0.033	0.024	6.14	0.013
4–10	CCGCGCC	0.026	0.031	0.022	5.97	0.015
3–9	CCCGCGC	0.027	0.033	0.023	6.88	0.009
2–8	ACCCGCG	0.028	0.033	0.025	4.53	0.033
3–8	CCCGCG	0.027	0.032	0.024	5.23	0.022
3–7	CCCGC	0.029	0.034	0.025	5.33	0.021
5–9	CGCGC	0.030	0.035	0.026	5.08	0.024

The table shows selected haplotypes associated with the disease. The most frequent 10-mer haplotype (0.14) was employed as the baseline to determine the relative effects of the other haplotypes. The studied SNPs are rs2740434 (also denoted as 1), rs17153743 (2), rs13264774 (3), rs56298569 (4), rs804280 (5), rs3729855 (6), rs3729856 (7), rs1062219 (8), rs11785481 (9) and rs3203358 (10) arranged sequentially by their chromosomal positions, whereby blocks represent the range of variants constituting the respective haplotypes. *p < 0.01; **p < 0.005 by *χ*^2^ test.

### GATA4 haplotyping and cardiovascular disease traits

Since several SNPs were associated with the different cardiovascular disease traits, we were interested in testing the likelihood of these relationships being delineable at the haplotype level. We employed the most frequent 10-mer GACACACCCG (frequency = 0.145) as the baseline for the analyses. While the 9-mer haplotype, ACCCGTGCC (*χ*^2^ = 3.80; *p* = 0.051) was weakly associated with CAD, its 8-mer derivative CCCGTGCC (*χ*^2^ = 4.71; *p* = 0.041) was associated with the disease. This relationship became stronger with the shortening of these haplotype sequences, culminating in the 4-mer ACCC (*χ*^2^ = 7.17; *p* = 0.007) displaying the most significant relationship with disease (Table 3; Additional file 2, GATA4 Haplo Suppl data). A similar trend was observed for the 8-mer GACCCGTG (*χ*^2^ = 3.84; *p* = 0.050) which also culminated in the 4-mer GACC (*χ*^2^ = 5.69; *p* = 0.017) showing the most significant association with the disease. Put together, these trends indicate that the SNPs rs17153743 (2), rs13264774 (3), rs56298569 (4) and rs804280 (5) constituted the primary core for this association with CAD. Myocardial infarction was equipotently linked to the 8-mer ACCCACGC (*χ*^2^ = 6.58; *p* = 0.010) and its 7-mer derivative ACCCACG (*χ*^2^ = 6.76; *p* = 0.009), thereby sharing with CAD, the same three causative nucleotides of the four SNPs comprising the core of the relationships. Notable relationships also included the 10-mer AACACATCCC (*χ*^2^ = 6.01; *p* = 0.014) and the 4-mer ATAC (*χ*^2^ = 7.98; *p* = 0.005) showing the most significant protective property against acquiring MI.

**Table 2:** Association of GATA4 gene variants with metabolic disease risk traits

**Block**	**Haplotype**	**Pooled**	**Cases**	**Control**	***χ*** **2**	***p*** **value**
Coronary artery disease						
1–8	GACCCGTG	0.108	0.114	0.101	3.84	0.050
3–10	CCCGTGCC	0.110	0.116	0.102	4.17	0.041
2–9	ACCCGTGC	0.112	0.119	0.105	3.87	0.049
	ACCCATGC	0.026	0.029	0.022	4.08	0.044
1–7	GACCCGT	0.109	0.116	0.102	3.93	0.048
	CCCATGC	0.026	0.029	0.022	3.81	0.050
2–8	ACCCGTG	0.113	0.120	0.105	4.51	0.034
4–10	CCGTGCC	0.111	0.118	0.104	3.91	0.048
4–9	ACATCC	0.069	0.064	0.075	4.60	0.032
3–8	CCCGTG	0.114	0.121	0.106	4.63	0.031
2–7	ACCCGT	0.119	0.126	0.111	4.66	0.031
3–7	CCCGT	0.119	0.126	0.110	4.96	0.026
1–5	GACCC	0.239	0.250	0.228	5.48	0.019
4–8	CCGTG	0.116	0.122	0.108	4.21	0.040
1–4	GACC	0.244	0.254	0.232	5.69	0.017
4–7	CCGT	0.121	0.129	0.113	4.73	0.030
2–5	ACCC	0.403	0.416	0.387	7.17	0.007*
Myocardial infarction						
1–10	AACACATCCC	0.04	0.038	0.049	6.01	0.014
1–9	AACACATCC	0.041	0.038	0.050	6.33	0.012
1–8	AACACATC	0.042	0.038	0.050	6.54	0.011**
2–9	ACCCACGC	0.029	0.033	0.023	6.58	0.010**
3–9	CCCACGC	0.029	0.032	0.023	5.97	0.015
2–8	ACCCACG	0.033	0.037	0.026	6.76	0.009**
6–10	GTCCC	0.014	0.016	0.011	3.84	0.050
4–8	CCACG	0.034	0.038	0.028	5.60	0.018
4–8	ATAC	0.013	0.010	0.018	7.98	0.005**
2–5	ACCC	0.403	0.410	0.387	4.07	0.044

The table shows selected haplotypes associated with disease. The most frequent 10-mer haplotype (0.14) was employed as the baseline to determine the relative effects of the other haplotypes. The studied SNPs are rs2740434 (also denoted as 1), rs17153743 (2), rs13264774 (3), rs56298569 (4), rs804280 (5), rs3729855 (6), rs3729856 (7), rs1062219 (8), rs11785481 (9) and rs3203358 (10) arranged sequentially by their chromosomal positions, and blocks represent the range of variants constituting the respective haplotypes. *p < 0.01; **p < 0.005 by *χ*^2^ test.

Since the outgoing notion was that GATA4 may mediate dyslipidaemia-mediated onset of CAD, it was interesting to investigate whether haplotyping might reveal any link with lipidaemic disorders. Interestingly, hChol (*χ*^2^ = 3.86; *p* = 0.050) and elevated hLDLC levels (*χ*^2^ = 4.94; *p* = 0.026) shared the common 9-mer AACCCATGT, while the 10-mer AACCCATGTC was associated with hLDLC (*χ*^2^ = 4.83; *p* = 0.028), but only weakly so with hChol (*χ*^2^ = 3.24; *p* = 0.072) (Table 4; Additional file 2, GATA4 Haplo Suppl data). Hypertriglyceridaemia (hTG) was linked to the 5-mer ACCTA (*χ*^2^ = 4.65; *p* = 0.031), while low high-density lipoprotein was associated with the CCCAC (*χ*^2^ = 6.54; *p* = 0.011).

**Table 3:** Association of GATA4 haplotypes with coronary artery disease/myocardial infarction

**Variant**	**Genotype/allele**	**Controls**	**Cases**	**Univariate analysis**		**Multivariate analysis**	
				***p*** **value**	**Exp (B)(95% CI)**	**Corrected** ***p*** **value**	**Exp (B″)(95% CI)**
Hypercholesterolaemia							
rs804291CT	TT	0.016	0.027	0.023*	1.66(1.07–2.57)	0.037*	1.61(1.03–2.52)
rs11785481CT	CT + TT	0.196	0.220	0.065	1.16(0.99–1.34)	0.087	1.15(0.98–1.35)
rs3203358CG	CG + GG	0.358	0.323	0.020*	0.85(0.75–0.98)	0.026*	0.86(0.75–0.98)
Hypertriglyceridaemia							
rs3729855CT	T	0.030	0.041	0.023	1.35(1.01–1.78)	0.004**	1.49(1.14–1.94)
	CT + TT	0.053	0.070	0.005**	1.34(1.09–1.64)	0.010*	1.47(1.10–1.96)
rs1062219CT	TT	0.220	0.182	0.011*	0.79(0.67–0.98)	0.016*	0.80(0.67–0.99)
Elevated low-density lipoprotein-cholesterol							
rs804291CT	TT	0.018	0.033	0.016*	1.90(1.13–3.21)	0.020*	1.87(1.10–3.15)
rs11785481CT	CT + TT	0.201	0.229	0.032*	1.18(1.01–1.37)	0.136	1.18(0.95–1.45)
Low high-density lipoprotein-cholesterol							
rs2740434CT	CT + TT	0.084	0.069	0.013*	0.81(0.69–0.96)	0.744	0.93(0.62–1.41)
Hypertension							
rs3729855CT	T	0.027	0.035	0.089	1.30(0.95–1.77)	0.013*	1.52(1.09–2.11)
rs17153743AG	AG + GG	0.017	0.031	0.022*	1.87(1.10–3.18)	0.005*	2.30(1.30–4.26)
rs13264774CT	T	0.041	0.024	0.008*	0.59(0.40–0.87)	0.085	0.68(0.44–1.05)
Type 2 diabetes mellitus							
rs3729855CT	T	0.040	0.028	0.003*	0.70(0.55–0.89)	0.003*	0.68(0.53–0.88)
rs13264774CT	CT + TT	0.035	0.023	0.025*	0.66(0.045–0.95)	0.199	0.76(0.51–1.16)
Obesity							
rs17153743AG	GG	0.138	0.070	0.031	0.73(0.55–0.98)	0.357	0.94(0.82–1.07)
rs11785481CT	TT	0.029	0.022	0.033*	0.73(0.55–0.98)	0.903	0.84(0.73–0.95)
rs2740434GA	GA + AA	0.086	0.065	0.013*	0.73(0.50–0.94)	0.011*	0.72(0.56–0.93)

The table summarizes the univariate and multivariate analyses for the relationships of the gene variants with various cardiovascular disease traits. Bonferroni tests have been performed to adjust for age and sex and adjustment made for the confounders in the multivariate tests. *p < 0.05; **p < 0.005.

We then proceeded to investigate further potential relationships of these haplotypes with other metabolic risk traits, particularly hypertension, type 2 diabetes and obesity. The results showed that a 10-mer GACCCGCGCC (*χ*^2^ = 7.59; *p* = 0.006) was associated with obesity, while a 9-mer GACACACCC (*χ*^2^ = 4.05; *p* = 0.044) was implicated in type 2 diabetes mellitus (Table 4). We also observed several protective haplotypes for all disease traits, which were primarily complementary to the core of the causative sequences (See also Additional file 2, GATA4 Haplo Suppl data). However, we could not establish any significant causative link with hypertension.

## Discussion

In the present study, we first screened two families with HFH in which the primary probands presented with severe phenotypes of early onset CAD. We identified several potential loci of which Chr 8 appealed as the most attractive choice for detailed investigation on the genetic basis for dyslipidaemia-related onset of the disease. This locus harbours the *GATA4* gene at Chr 8p23.1-22, which we elected to study first as the most likely candidate. Screening the complete coding and non-coding areas of the gene revealed several mutations, among which we selected 12 for the case-control study. The study has unequivocally identified two SNPs, the rs1062219 and rs804280 as risk variants for CAD, both of which were also linked to congenital heart disease in the present study as well as other previous studies in different ethnic populations [25,26,30]. Therefore, our study does not only furnish strong support for an important role for these SNPs in congenital heart disease, but also points to a possible sharing of common disease pathways involved in the etiologies of CAD and congenital heart disease at the GATA4 signaling level. Notably, by far, the majority of GATA4 mutations studied to date seem to point to their influencing its transcriptional activity, and have been associated primarily with cardiac malformations, such as congenital heart disease. Thus, the difference in the nature of the congenital heart disease and CAD etiologies renders it very intriguing why these two disorders would share the *GATA4*, or any other signalling pathway for that matter, as a common disease pathway. A number of speculations have recently been advanced on how changes in GATA4 gene may influence disease pathways. One of these suggests an impediment of the GATA4 transcription activity in congenital heart disease as involving at least two of the variants, p.A348A and p.S377G [26], which were also included in the present study. Since GATA4 transcription activity is subject to regulation at the level of gene expression and through post-translational modifications of protein, the processes involved in these regulatory mechanisms are therefore worthy considering as possible culprits in our study. On the other hand, however, in our study, while the former was implicated in MI, the latter was actually protective against acquiring CAD. Accordingly, these observations seem to point to delineable differences between CAD and congenital heart disease in their interactions with the *GATA4* variants. Besides, the ubiquity of GATA4 in the myocardium also raises fundamental questions with respect to possible diversity in its cardiac-related function(s) and its involvement in other cardiovascular disease pathways, such as those leading to atherosclerosis. Hence, the primary question raised in our study was whether a link existed between the impact of GATA4 on CAD manifestation and the presence of metabolic disorders, as our linkage study in HFH seemed to suggest.

To begin with, our results pointed to an association of the rs804291 and rs11785481 with both hChol and hLDLC, which appeared to follow primarily an autosomal recessive mode of inheritance. A closer analysis of the data also indicated that while the rs11785481 was not directly related to CAD per se, the probability of an individual acquiring CAD increased greatly in hypercholesterolaemic individuals. We also noted that two other SNPs that were not directly associated with hChol became so in the presence of MI, similarly indicative of a possible interaction of the dyslipidaemic disease traits with changes in GATA4 as a possible link to CAD/MI manifestation in these individuals. Moreover, the analyses for the other metabolic risk factors revealed the association for two variants with hypertension, further linking risk traits for metabolic syndrome to GATA4 polymorphism. Altogether, these observations furnish support for the notion of a contribution of some interactions of metabolic risk traits with GATA4 to the disease pathways leading to atherosclerosis, an assertion which requires further investigation.

The various ways in which the *GATA4* variants relate to alterations in lipid levels and CAD/MI in this study lead to important questions regarding the possible mechanisms or pathways linking them with one another. Based on our results, it appears that such mechanisms may involve events associated with the harbouring of lHDLC, for example, being linked to pathways directly influencing circulating lipid levels and possibly leading to acquiring CAD in dyslipidaemic individuals, as indicated by the inverse relationship of some of these traits with the different *GATA4* variants. Accordingly, the processes appear to be separable from those leading to cardiac malformations. Hence, the novel finding implicating the *GATA4* in dyslipidaemia seems to point to yet undefined entities, possibly involving the regulation of the GATA4 functional state, as playing an important role in the disease process. In this regard, perhaps the most revealing observation of the present study is the linking of non-coding variants of the gene to disease. Of particular interest was the finding of causative haplotypes for CAD/MI as well as the metabolic risk traits encompassing variants in the 3′-UTR of the gene. Thereby, isolating the two coding SNPs from those residing in the 3′-UTR did not alter the significance level for the latter, possibly pointing to the changes at this chromosomal locus rather than the individual variants as the underlying genetic basis for these manifestations. Notably, the significance levels for the haplotype associations were higher than those of individual constituent variants, pointing to the importance of these genomic sequences in revealing the impact of a gene on disease that would otherwise remain uncovered through associations with changes at individual loci. Implications are that these events may be due to changes other than those directly pertaining to a functional motif of the GATA4 protein. Furthermore, the close proximity of these variants may also suggest the presence of sequences encoding some other yet unidentified entities as the potential culprits, especially considering the fact that several 3-mer haplotypes at this locus were shared by some of the traits. Thus, it is plausible to postulate that alteration at this genomic locus, and not necessarily in the *GATA4* gene function per se, may offer an explanation for the observed link to alterations in the metabolic risk traits and CAD manifestation.

A number of speculations have been raised with regard to the possible ways in which changes in the 3′-UTR of the *GATA4* gene may influence disease pathways. For example, both germline and somatic mutations have recently been described in this region that were predicted to affect RNA folding as cause for congenital heart disease [25]. This is in line with the notion that the mechanisms by which *GATA4* contributes to hChol metabolism may be related to the regulation of the gene itself or its mRNA maturation rather than the transcriptional activity of its protein product. These mechanisms are likely to be the result of complex interactions with yet unidentified co-factors, as has been suggested recently by some studies [6]. While the study has produced interesting data that may contribute to our knowledge of GATA4 interaction with cardiovascular disease, there are some limitations in the extent of its applicability. Thus, one of the main limitations is that no such potential mechanisms were tested to verify the notion that the 3′-UTR of the *GATA4* gene plays an important role in the discussed cardiovascular disease pathways. Furthermore, like other association studies on complex diseases, the potential impact of the present findings may be limited to ethnic Arab populations due to inter-ethnic variations in prevalent epigenetic and environmental factors. Besides, it is not likely that our present findings per se can be exploited as predictive markers for the dyslipidaemic disorders.

## Conclusion

In summary, our study identified the GATA4 transcription factor as an independent risk factor for congenital heart disease and CAD/MI and a metabolic risk trait for cardiovascular diseases. Thus, apart from the demonstrated association of *GATA4* polymorphism with dyslipidaemia, our results also point to interrelationships of the two disease components with CAD/MI, which may explain partly how these diseases lead to atherosclerosis. The finding of several causative haplotypes for MI/CAD embracing the 3′UTR of the *GATA4* gene points to important roles for this chromosomal locus in the etiology of CAD/MI and the possible involvement of yet undefined entities related to GATA4 transcription activity or gene regulatory pathways in events leading to these cardiovascular disorders.

## Materials and methods

### Study population

**Figure 3:**
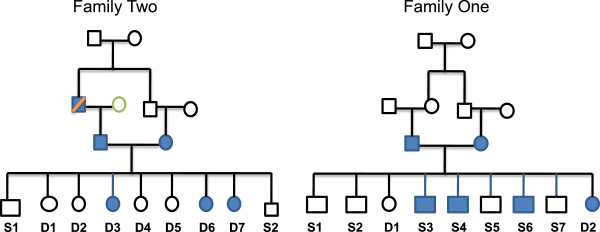
**Pedigrees of two families with heterozygous hyperlipidaemia.** Proband S3 in Family One underwent triple coronary artery bypass at the age of 14 years, as well as D6 and D7 in Family Two presented with early onset coronary artery disease at the age of 17 and 15 years.

The present study was performed in three stages involving three independent groups of Saudi individuals. The first group comprised two families of a total of 22 members in which HFH was prevalent (Figure 3). The index case of the first family was the third (S3) of seven sons and two daughters born to unrelated parents, who underwent triple coronary artery bypass surgery at the age of 14 years. On admission, this propositus had reported with a chest wound and xanthomas, as well as clinical features of bilateral carotid artery disease and a very severe form of familial hyperlipidaemia (FH). He presented with very high cholesterol (Chol) level of 10.1 mmol/L (desirable <5.2 mmol/l) and LDL-Chol level of 7.9 mmol/L (optimal <2.59 mmol/l). Furthermore, he harboured low HDL-Chol levels (0.51 mmol/l; normal 1.04–1.55). The father displayed the characteristics of borderline HFH (Chol 6.1 mmol/L, LDL 4.0 mmol/L), while the mother, two other sons (S4 and S6) and a daughter (D2) had clinical phenotypes of the disease (combination of high Chol (≥6.2 mmol/L) and high LDL-Chol (≥4.12 mmol/L)). Two other sons with otherwise normal Chol and LDL-Chol levels also displayed low HDL-Chol levels (<1.04 mmol/L), while none of the family members had isolated elevated LDL-Chol levels. In the second family, two index cases, two daughters of a family of seven daughters and two sons concurrently presented with early onset CAD at the age of 17 and 15 years. The two candidates had identical exceedingly high Chol levels of 22.5 mmol/l and LDL-Chol levels of 19.6 (D6) and 18.0 mmol/l (D7), in addition to harbouring low HDL levels of 0.93 and 0.65 mmol/l, respectively. The father (8.6 mmol/l), mother (7.7 mmol/l) and a third daughter (D3; 7.6 mmol/l) were also hypercholesterolaemic. The criteria for the diagnosis of familial hyperlipidaemia (FH) were adapted from our Institutional Guidelines, employing the reference values approved by the USA National Cholesterol Education Program (NCEP). Following the identification of Chr 8p23 region, which harbours the *GATA4* gene (8p23.1-22), as a potential culprit for the disease through linkage study of the above two families, we elected to sequence the gene in the two families and a group of 250 healthy blood donors visiting our Blood Bank Clinic to identify potentially informative SNPs of interest for the case-control association study.

The case-control study was performed in a total of 4,274 Saudi individuals consisting of 2,274 CAD patients (1,736 males and 538 females, mean age 60.8 ± 0.4 years) with angiographically determined narrowing of the coronary vessels by at least 50% and 2,004 angiographed controls (1,074 male and 930 female, mean age 50.2 ± 0.5 years) (Table 5). These controls (CON) were individuals undergoing surgery for valvular disease or reporting for follow-up on various other cardiac procedures, but were established to have no significant narrowing of the coronary vessels. Among the study population, 1,590 patients were hypercholesterolaemic (hChol) patients (1,051 male and 539 female, mean age 58.2 ± 0.2 years) undergoing anti-hyperlipidaemic therapy and/or known to have high levels of total cholesterol (Chol >6.20 mmol/L), 1,776 patients harboured lHDLC levels (<1.04 mmol/L), 1,088 had high triglyceride (hTG) levels (>2.25 mmol/L), and 575 exhibited high low-density lipoprotein levels. These were denoted as dyslipidaemia study patients. Another subset of interest comprised 2,378 individuals having type 2 diabetes mellitus (T2DM; formerly known as non-insulin-dependent diabetes mellitus or adult-onset diabetes). The National Diabetes Data Group of the USA and the second World Health Organization Expert Committee on Diabetes Mellitus (1998) [31] define type 2 diabetes mellitus as a metabolic disorder that is characterized by high blood glucose (generally defined as fasting glucose level >126 mg/dL) in the context of insulin resistance and relative insulin deficiency. Furthermore, 3,312 individuals had primary (essential) hypertension (HTN) (Table 5), defined as ≥140 mm Hg systolic blood pressure or ≥90 mm Hg diastolic pressure based on The Sixth Report of the Joint National Committee on Prevention, Detection, Evaluation, and Treatment of High Blood Pressure (JNC VI) criteria [32]. Accordingly, essential, primary, or idiopathic hypertension is defined as high blood pressure in which secondary causes such as renovascular disease, renal failure, pheochromocytoma, aldosteronism, or other causes of secondary hypertension or Mendelian forms (monogenic) are absent [32]. The fourth group comprised 1,631 obese candidates with body-mass index (BMI) of ≥30.0 kg/m^2^, and classified as the obesity subset. Among these subsets of patients, some patients harboured a combination of two or possibly three of the cardiovascular risk traits. All individuals with major cardiac rhythm disturbances, incapacitating or life-threatening illness, major psychiatric illness or substance abuse, history of cerebral vascular disease, neurological disorders, and administration of psychotropic medication or any other disorders likely to interfere with variables under investigation were excluded from the study. This study was performed in accordance with the regulations laid down by the King Faisal Specialist Hospital and Research Centre Ethics Committee in compliance with the Helsinki Declaration [33] and all participants signed an informed consent.

**Table 4:** Association of GATA4 haplotypes with metabolic disease risk traits

	**Controls**			**Cases**		
	**All**	**Male**	**Female**	**All**	**Male**	**Female**
Gender	2,004	1,074 (53.6)	930 (46.4)	2,274	1,736 (76.3)	538 (23.7)
Age	50.2 ± 0.5	50.81 ± 0.5	49.66 ± 0.5	60.8 ±0.4	59.7 ± 0.3	61.8 ± 0.5
BMI	29.4 ±0.2	28.01 ± 0.2	30.69 ± 0.2	29.7 ± 0.2	28.3 ± 0.1	31.1 ± 0.3
MI	1,388	684 (0.49)	704 (0.51)	2,890	2,126 (0.74)	764 (0.26)
T2DM	1,900	1,220 (0.64)	680 (0.36)	2,378	1,590 (0.67)	788 (0.33)
HTN	966	650(0.67)	314 (0.33)	3,312	2,158 (0.65)	1,154 (0.35)
lHDLC	2,209	1,262 (0.57)	947 (0.43)	1,776	1,371 (0.77)	405 (0.23)
hLDL	3,404	2,267 (0.67)	1,137 (0.33)	575	364 (0.63)	211 (0.37)
hTG	2,896	1,859 (0.64)	1,037 (0.36)	1,088	773 (0.71)	315 (0.29)
hChol	2,487	1,639 (0.66)	848 (0.34)	1,590	1,051 (0.66)	539 (0.34)
CHD	4,128	2,741 (0.66)	1,387 (0.34)	150	69 (0.46)	81 (0.54)
FH	3,421	2,255 (0.66)	1,166 (0.34)	857	555 (0.65)	302 (0.35)
OBS	2,362	1,729 (0.73)	633 (0.27)	1,631	895 (0.55)	736 (0.45)
Smokers	2,575	1,223 (0.47)	1,352 (0.53)	1,619	1,547 (0.96)	72 (0.04)
VD						
One	0	0	0	847	614 (0.72)	233 (0.28)
Two	0	0	0	456	355 (0.78)	101 (0.22)
More than two	0	0	0	971	767 (0.79)	204 (0.21)

The numbers in brackets give the percentages of the total (all) values of the group. BMI, body mass index; CHD, congenital heart disease; FH, family history of CAD; MI, myocardial infarction; lHDLC, low high-density lipoprotein-cholesterol level; hTG, hypertriglyceridaemia; hChol, hypercholesterolaemia; HTN, hypertension; T2DM, type 2 diabetes mellitus; VD, number of diseased vessels.

**Table 5:** Patient Demographics and clinical data

**Variant**	**Genotype/allele**	**Controls**	**Cases**	**Univariate analysis**		**Multivariate analysis**	
				***p*** **value**	**Exp (B)(95% CI)**	**Corrected** ***p*** **value**	**Exp (B″)(95% CI)**
Coronary artery disease							
rs3729855CT	T	0.037	0.030	0.047*	0.78(0.62–1.00)	0.315	0.87(0.67–1.14)
rs3203358CG	G	0.206	0.191	0.074	0.90(0.82–1.00)	0.042*	0.88(0.78–0.99)
rs1062219CT	CT + TT	0.663	0.694	0.034*	1.15 (1.01–1.31)	0.091	1.13(0.98–1.31)
rs17153743AG	AG + GG	0.033	0.022	0.036*	0.67(0.47–0.98)	0.032*	0.67(0.43–0.96)
rs804280AC	C	0.420	0.446	0.018*	1.11(1.02–1.21)	0.009*	1.14(1.03–1.27)
	AC + CC	0.647	0.688	0.005**	1.20(1.06–1.36)	0.012*	1.20(1.03–1.39)
Myocardial infarction							
rs3729855CT	T	0.040	0.030	0.020*	0.75(0.59–0.96)	0.323	0.86(0.86–1.56)
rs3729856AG	GG	0.031	0.041	0.024*	1.34(1.04–1.72)	0.146	1.42(0.88–2.33)
rs13264774CT	CT + TT	0.269	0.247	0.029*	0.89(0.80–0.99)	0.103	0.84(0.69–1.04)
rs804280AC	AC + CC	0.645	0.681	0.020*	1.17(1.07–1.29)	0.511	1.07(0.88–1.28)
Congenital heart disease							
rs3729856AG	G	0.145	0.215	0.012*	1.61(1.11–2.30)	0.081	1.51(0.95–2.40)
	AG + GG	0.280	0.362	0.018*	1.45(1.07–1.99)	0.108	1.57(0.90–2.73)
rs12825CG	CG + GG	0.777	0.854	0.010*	1.68(1.13–2.50)	0.108	1.71(0.89–3.29)
rs11785481CT	T	0.115	0.154	0.037*	1.41(1.02–1.94)	0.030*	1.78(1.06–3.00)
	CT + TT	0.190	0.275	0.053*	1.61(1.15–2.27)	0.183	1.50(0.83–2.75)
rs2740434CT	CT + TT	0.073	0.123	0.020*	1.78(1.09–2.89)	0.403	0.70(0.30–1.63)
rs13264774CT	CT + TT	0.267	0.205	0.057	0.71(0.049–1.01)	0.238	0.69(0.38–1.27)

The table summarizes the univariate and multivariate analyses for the relationships of the gene variants with coronary artery disease, myocardial infarction and congenital heart disease in the studied 4,278 individuals. Bonferroni tests have been performed to adjust for age and sex, and adjustment made for the confounders in the multivariate tests.*p < 0.05; **p < 0.005.

Five millilitres of peripheral blood was sampled in EDTA tubes after obtaining written consent, and genomic DNA extracted from leukocytes by the standard salt methods using PUREGENE DNA isolation kit (Qiagen, Germantown, MD, USA).

### Linkage analysis and gene sequencing

Whole genome-wide scanning of two families with heterozygous familial hypercholesterolaemia was performed using the Affymetrix Gene Chip 250 Sty1 mapping array (Affymetrix, Inc., Santa Clara, CA, USA), and multipoint parametric linkage analysis for estimating the LOD scores performed using the GeneHunter Easy Linkage analysis software 4.0 (Affymetrix, Inc., Santa Clara, CA, USA) as described previously [34]. A recessive model of inheritance was used with a population-disease allele frequency of 0.0001, based on the Asian SNP allele frequencies, and the Copy Number Analyzer for GeneChip® (CNAG) Ver. 3.0 (Affymetrix, Inc., Santa Clara, CA, USA) software was employed in order to search for shared chromosomal regions of homozygosity. Following the identification of Chr 8 as a potential risk locus, screening for GATA4 mutations of interest was accomplished by sequencing on the MegaBACE DNA analysis system (Amersham Biosciences, Sunnyvale, CA, USA). Briefly, the DNA was subjected to PCR amplification by standard methods, following which the PCR products were sequenced and the data analyzed for SNPs by Lasergene software (DNASTAR, Inc. Madison, WI, USA) as described previously [35].

### Association studies

Following the identification of possible informative variants based on the screening of our general population, genotyping was accomplished using Taqman chemistry on the Applied Biosystems Prism 7900HT Sequence Detection System (ABI Inc., Foster City, CA, USA). Primers and the TaqMan probes were procured from Applied Biosystems (ABI, Warrington, UK), and assays run by standard methods (Eurogentec, Seraing, Belgium) on the ABI2720 thermocycler (ABI Inc., Foster City, CA, USA). The plates were then scanned for FRET signal and data analyzed using Applied Biosystems SDS 2.4 software.

### Statistical analysis

Comparison of genotypes and alleles between different groups for continuous dependent variables was achieved by Analysis of Variance (ANOVA) or Student's *t* test as appropriate. Categorical variables were analyzed by Chi-square test, and logistic regression analysis was used to compute odds ratios and their 95% confidence intervals. The Bonferroni test was employed to correct for the potential impact of the classical confounders, such as age, sex, smoking and disease history for all studied disease traits. Initially, univariate analysis was performed for individual SNPs to test for their possible association with the different traits. For the SNPs, that showed a significant value at *p* < 0.05, we then performed two tests, multivariate and multinomial regression analyses to test for possible confounding effects of the different disease traits in the model. The Haplo.stats package [36] in the R Statistical Computing software [37] was used to perform haplotype-based association analysis. Odd ratios for haplotypes were calculated using the baseline 10-mer haplotype GACACACCCG (global score statistics = 14.4) as reference, and the Haplotype Score statistic for the association of a haplotype with the binary trait was calculated as in [38] and [39]. Significance of association was determined between haplotypes and the case-control status - a binomial trait denoting whether or not a patient had the disease. All other statistical analyses were performed using the SPSS software Version 20 (SPSS Inc., Chicago, IL, USA), and data are expressed as mean ± SEM. Associations with a two-tailed *p* value < 0.05 was considered statistically significant.

## Abbreviations

CAD: coronary artery disease; CHD: congenital heart disease; CBP: cAMP-response element-binding (CREB) protein; FH: family history of CAD; GATA4: GATA binding protein 4; hChol: hypercholesterolaemia; HFH: familial hypercholesterolaemia; hLDLC: high low-density lipoprotein-cholesterol; hTG: hypertriglyceridaemia; HTN: hypertension; lHDLC: low high-density lipoprotein-cholesterol level; MI: myocardial infarction; T2DM: type 2 diabetes mellitus; TFs: transcription factors

## Competing interests

The authors declare that they have no competing interests.

## Authors' contributions

NPM was involved in running the Affymetrix assays, designing probes, screening for gene mutations as well as participating in the write-up of the manuscript. SMW performed Affymetrix genotyping analysis. AIT performed the statistical analysis. SH ran the Affymetrix assays. MN and DG were responsible for the overall running of the Taqman assays. NAT contributed to statistical analysis. EA was responsible for procuring patient material and data. NM was responsible for the clinical patient data and material acquisition. BFM contributed to the write-up of the manuscript. ND is the Principal Investigator, with the overall responsibility for the project and preparation of the manuscript. All authors read and approved the final manuscript.

## Supplementary Material

Additional file 1 GATA4 Suppl data: analysis for GATA4 interaction with age, sex and other confounders for the analyses of the different disease traits. Click here for file

Additional file 2 GATA4 Haplo Suppl data: GATA4 haplotyping for the cardiovascular disease traits. Click here for file
