# Supplementary material for: A study of the role of GATA4 polymorphism in cardiovascular metabolic disorders
Source: Hum Genomics. 2013 Dec 12;7(1):25. doi: 10.1186/1479-7364-7-25 (PMC3899629; doi:10.1186/1479-7364-7-25)
Supplement: Additional file 1 — GATA4 Suppl data: analysis for GATA4 interaction with age, sex and other confounders for the analyses of the different disease traits. [file 1479-7364-7-25-S1.doc]

**GATA4 Suppl data 01:** Analysis for GATA4 Interaction with age, sex and other confounders for the analyses of the different disease traits. The analysis includes the Bonferroni corrections for each data set.

**SNP Interaction with Hypertension**

| Model | Unstandardized Coefficients | | Standardized Coefficients | t | P-value | 95.0% Confidence Interval for B | |
| --- | --- | --- | --- | --- | --- | --- | --- |
|  | B | Standard Error | Beta |  |  | Lower Bound | Upper Bound |
| (Constant) | .357 | .137 |  | 2.611 | .009 | .089 | .625 |
| Sex | .034 | .010 | .039 | 3.407 | .001 | .014 | .053 |
| MI | .051 | .011 | .057 | 4.708 | .000 | .030 | .072 |
| FH | -.019 | .011 | -.018 | -1.654 | .098 | -.041 | .004 |
| T2DM | .157 | .010 | .188 | 15.983 | .000 | .138 | .176 |
| OBS | .063 | .009 | .074 | 6.779 | .000 | .044 | .081 |
| HTG | -.004 | .011 | -.004 | -.344 | .731 | -.024 | .017 |
| LHDL | .010 | .009 | .011 | 1.022 | .307 | -.009 | .028 |
| HChol | .063 | .010 | .074 | 6.193 | .000 | .043 | .083 |
| HLDL | -.009 | .014 | -.007 | -.648 | .517 | -.035 | .018 |
| Age (Binned) | .062 | .003 | .211 | 17.829 | .000 | .055 | .069 |
| rs3729855C | -.154 | .167 | -.070 | -.927 | .354 | -.481 | .172 |
| rs3729855T | -.087 | .169 | -.037 | -.518 | .604 | -.418 | .243 |
| rs3729856AA | .146 | .270 | .135 | .541 | .589 | -.384 | .676 |
| rs3729856G | .165 | .271 | .151 | .611 | .541 | -.365 | .696 |
| rs1062219C | -.041 | .145 | -.049 | -.282 | .778 | -.325 | .243 |
| rs1062219T | -.032 | .145 | -.039 | -.222 | .824 | -.316 | .251 |
| rs12825C | -.107 | .080 | -.128 | -1.338 | .181 | -.264 | .050 |
| rs12825G | -.119 | .080 | -.143 | -1.496 | .135 | -.275 | .037 |
| rs804291AA | .271 | .118 | .326 | 2.285 | .022 | .038 | .503 |
| rs804291G | .277 | .118 | .333 | 2.353 | .019 | .046 | .508 |
| rs11785481C | -.283 | .164 | -.221 | -1.723 | .085 | -.605 | .039 |
| rs11785481T | -.286 | .165 | -.220 | -1.734 | .083 | -.609 | .037 |
| rs3203358C | .437 | .181 | .424 | 2.408 | .016 | .081 | .792 |
| rs3203358G | .446 | .182 | .429 | 2.454 | .014 | .090 | .802 |
| rs2740434G | -.022 | .120 | -.023 | -.182 | .856 | -.256 | .213 |
| rs2740434AA | -.011 | .120 | -.012 | -.092 | .927 | -.246 | .224 |
| rs17153743AA | -.180 | .117 | -.053 | -1.534 | .125 | -.410 | .050 |
| rs17153743G | -.068 | .124 | -.018 | -.551 | .581 | -.312 | .175 |
| rs13264774C | -.013 | .026 | -.011 | -.479 | .632 | -.065 | .039 |
| rs13264774T | -.018 | .029 | -.014 | -.604 | .546 | -.075 | .040 |
| rs804280AA | -.029 | .090 | -.035 | -.326 | .744 | -.205 | .147 |
| rs804280C | -.056 | .090 | -.066 | -.620 | .535 | -.231 | .120 |

SNP Interaction with myocardial infarction

| Model | Unstandardized Coefficients | | Standardized Coefficients | t | P-value | 95.0% Confidence Interval for B | |
| --- | --- | --- | --- | --- | --- | --- | --- |
|  | B | Standard Error | Beta |  |  | Lower Bound | Upper Bound |
| (Constant) | .343 | .120 |  | 2.854 | .004 | .108 | .579 |
| Sex | -.078 | .009 | -.078 | -8.888 | .000 | -.095 | -.061 |
| FH | -.044 | .010 | -.037 | -4.335 | .000 | -.063 | -.024 |
| T2DM | .085 | .009 | .091 | 9.943 | .000 | .069 | .102 |
| OBS | .020 | .008 | .021 | 2.426 | .015 | .004 | .036 |
| hTG | .006 | .009 | .006 | .628 | .530 | -.012 | .024 |
| lHDL | .033 | .008 | .035 | 4.056 | .000 | .017 | .049 |
| HChol | .029 | .009 | .030 | 3.195 | .001 | .011 | .047 |
| HLDL | -.007 | .012 | -.005 | -.554 | .579 | -.030 | .017 |
| CAD | .526 | .009 | .558 | 59.257 | 0.000 | .509 | .544 |
| Age (Binned) | .044 | .003 | .134 | 14.647 | .000 | .039 | .050 |
| rs3729855C | .358 | .147 | .144 | 2.446 | .014 | .071 | .646 |
| rs3729855T | .338 | .148 | .129 | 2.280 | .023 | .047 | .629 |
| rs3729856AA | -.763 | .238 | -.624 | -3.210 | .001 | -1.230 | -.297 |
| rs3729856G | -.755 | .238 | -.610 | -3.171 | .002 | -1.222 | -.288 |
| rs1062219C | .031 | .127 | .032 | .240 | .810 | -.219 | .281 |
| rs1062219T | .029 | .127 | .031 | .231 | .817 | -.220 | .279 |
| rs12825C | -.050 | .070 | -.053 | -.712 | .476 | -.188 | .088 |
| rs12825G | -.051 | .070 | -.055 | -.734 | .463 | -.189 | .086 |
| ra804291AA | -.070 | .104 | -.074 | -.670 | .503 | -.274 | .134 |
| ra804291G | -.066 | .104 | -.070 | -.637 | .524 | -.269 | .137 |
| rs11785481C | -.046 | .144 | -.032 | -.319 | .749 | -.329 | .237 |
| rs11785481T | -.073 | .145 | -.050 | -.501 | .616 | -.357 | .212 |
| rs3203358C | .302 | .160 | .260 | 1.894 | .058 | -.011 | .615 |
| rs3203358G | .297 | .160 | .253 | 1.858 | .063 | -.016 | .610 |
| rs2740434G | .065 | .105 | .061 | .618 | .537 | -.141 | .271 |
| rs2740434AA | .060 | .105 | .056 | .573 | .567 | -.146 | .267 |
| rs17153743AA | .064 | .103 | .017 | .622 | .534 | -.138 | .267 |
| rs17153743G | .062 | .109 | .015 | .570 | .569 | -.152 | .276 |
| rs13264774C | .043 | .023 | .034 | 1.827 | .068 | -.003 | .088 |
| rs13264774T | .034 | .026 | .025 | 1.334 | .182 | -.016 | .084 |
| rs804280AA | .009 | .079 | .009 | .108 | .914 | -.146 | .163 |
| rs804280C | .018 | .079 | .019 | .226 | .821 | -.137 | .172 |

**SNP Interaction with TYPE 2 DIABETES MELLITUS**

| Model | Unstandardized Coefficients | | Standardized Coefficients | t | P-value | 95.0% Confidence Interval for B | |
| --- | --- | --- | --- | --- | --- | --- | --- |
|  | B | Standard Error | Beta |  |  | Lower Bound | Upper Bound |
| (Constant) | -.012 | .160 |  | -.078 | .938 | -.326 | .301 |
| Sex | .043 | .012 | .041 | 3.733 | .000 | .021 | .066 |
| MI | .151 | .015 | .142 | 9.943 | .000 | .121 | .180 |
| FH | -.168 | .013 | -.136 | -12.698 | .000 | -.194 | -.142 |
| OBS | .094 | .011 | .093 | 8.730 | .000 | .073 | .115 |
| hTG | .115 | .012 | .103 | 9.395 | .000 | .091 | .139 |
| lHDL | .054 | .011 | .054 | 4.990 | .000 | .033 | .076 |
| hChol | .078 | .012 | .076 | 6.486 | .000 | .054 | .101 |
| hLDL | -.023 | .016 | -.017 | -1.480 | .139 | -.055 | .008 |
| CAD | .086 | .014 | .086 | 6.064 | .000 | .058 | .114 |
| Age (Binned) | .063 | .004 | .178 | 15.593 | .000 | .055 | .071 |
| rs3729855C | -.386 | .195 | -.146 | -1.983 | .047 | -.767 | -.004 |
| rs3729855T | -.474 | .197 | -.170 | -2.409 | .016 | -.860 | -.088 |
| rs3729856AA | -.028 | .316 | -.021 | -.087 | .930 | -.647 | .592 |
| rs3729856G | -.035 | .316 | -.026 | -.109 | .913 | -.655 | .586 |
| rs1062219C | -.058 | .169 | -.058 | -.343 | .732 | -.390 | .274 |
| rs1062219T | -.057 | .169 | -.057 | -.337 | .736 | -.388 | .274 |
| rs12825C | .263 | .093 | .263 | 2.817 | .005 | .080 | .446 |
| rs12825G | .261 | .093 | .262 | 2.808 | .005 | .079 | .443 |
| rs804291AA | -.283 | .138 | -.285 | -2.050 | .040 | -.555 | -.012 |
| rs804291G | -.287 | .137 | -.289 | -2.091 | .037 | -.557 | -.018 |
| rs11785481C | .544 | .192 | .355 | 2.835 | .005 | .168 | .919 |
| rs11785481T | .562 | .193 | .361 | 2.917 | .004 | .184 | .939 |
| rs3203358C | .297 | .212 | .241 | 1.402 | .161 | -.118 | .712 |
| rs3203358G | .301 | .212 | .242 | 1.418 | .156 | -.115 | .717 |
| rs2740434G | -.328 | .140 | -.292 | -2.352 | .019 | -.602 | -.055 |
| rs2740434AA | -.328 | .140 | -.289 | -2.342 | .019 | -.602 | -.053 |
| rs17153743AA | .066 | .137 | .016 | .479 | .632 | -.203 | .334 |
| rs17153743G | .177 | .145 | .040 | 1.224 | .221 | -.107 | .462 |
| rs13264774C | .055 | .031 | .041 | 1.781 | .075 | -.006 | .116 |
| rs13264774T | .046 | .034 | .032 | 1.362 | .173 | -.020 | .113 |
| rs804280AA | -.047 | .105 | -.047 | -.443 | .658 | -.252 | .159 |
| rs804280C | -.037 | .105 | -.037 | -.351 | .726 | -.242 | .169 |

**SNP Interaction with obesity**

| Model | Unstandardized Coefficients | | Standardized Coefficients | t | P-value | 95.0% Confidence Interval for B | |
| --- | --- | --- | --- | --- | --- | --- | --- |
|  | B | Standard Error | Beta |  |  | Lower Bound | Upper Bound |
| (Constant) | -.108 | .170 |  | -.632 | .527 | -.441 | .226 |
| Sex | .194 | .012 | .187 | 15.893 | .000 | .170 | .218 |
| T2DM | .106 | .012 | .107 | 8.730 | .000 | .082 | .130 |
| MI | .039 | .016 | .038 | 2.426 | .015 | .008 | .071 |
| FH | .111 | .014 | .091 | 7.846 | .000 | .084 | .139 |
| hTG | .015 | .013 | .013 | 1.124 | .261 | -.011 | .040 |
| lHDL | -.003 | .012 | -.003 | -.297 | .767 | -.026 | .019 |
| HChol | .085 | .013 | .084 | 6.691 | .000 | .060 | .111 |
| hLDL | -.055 | .017 | -.040 | -3.262 | .001 | -.088 | -.022 |
| CAD | -.053 | .015 | -.053 | -3.468 | .001 | -.082 | -.023 |
| Age (Binned) | -.002 | .004 | -.007 | -.543 | .587 | -.011 | .006 |
| rs3729855C | -.390 | .207 | -.149 | -1.880 | .060 | -.796 | .017 |
| rs3729855T | -.411 | .210 | -.149 | -1.961 | .050 | -.823 | .000 |
| rs3729856AA | .446 | .337 | .347 | 1.325 | .185 | -.214 | 1.106 |
| rs3729856G | .482 | .337 | .371 | 1.429 | .153 | -.179 | 1.142 |
| rs1062219C | .295 | .180 | .298 | 1.635 | .102 | -.059 | .648 |
| rs1062219T | .280 | .180 | .283 | 1.553 | .121 | -.073 | .633 |
| rs12825C | -.005 | .100 | -.005 | -.049 | .961 | -.200 | .190 |
| rs12825G | -.011 | .099 | -.011 | -.109 | .913 | -.205 | .184 |
| rs804291AA | .173 | .147 | .176 | 1.174 | .240 | -.116 | .462 |
| rs804291G | .179 | .147 | .182 | 1.222 | .222 | -.108 | .466 |
| rs11785481C | -.133 | .204 | -.088 | -.649 | .516 | -.533 | .268 |
| rs11785481T | -.126 | .205 | -.082 | -.612 | .540 | -.528 | .277 |
| rs3203358C | .039 | .226 | .032 | .172 | .863 | -.404 | .482 |
| rs3203358G | .048 | .226 | .039 | .214 | .830 | -.395 | .492 |
| rs2740434G | -.264 | .149 | -.237 | -1.774 | .076 | -.556 | .028 |
| rs2740434AA | -.258 | .149 | -.229 | -1.726 | .084 | -.550 | .035 |
| rs17153743AA | .089 | .146 | .022 | .607 | .544 | -.198 | .375 |
| rs17153743G | .121 | .154 | .027 | .780 | .435 | -.182 | .423 |
| rs13264774C | .008 | .033 | .006 | .232 | .817 | -.057 | .072 |
| rs13264774T | -.014 | .036 | -.010 | -.382 | .702 | -.085 | .057 |
| rs804280AA | -.100 | .112 | -.101 | -.894 | .371 | -.319 | .119 |
| rs804280C | -.106 | .112 | -.107 | -.953 | .341 | -.325 | .112 |

SNP Interaction with hypertriglyceridaemia

| Model | Unstandardized Coefficients | | Standardized Coefficients | t | P-value | 95.0% Confidence Interval for B | |
| --- | --- | --- | --- | --- | --- | --- | --- |
|  | B | Standard Error | Beta |  |  | Lower Bound | Upper Bound |
| (Constant) | -.095 | .149 |  | -.639 | .523 | -.388 | .197 |
| Sex | -.020 | .011 | -.021 | -1.791 | .073 | -.041 | .002 |
| OBS | .011 | .010 | .012 | 1.124 | .261 | -.008 | .031 |
| T2DM | .100 | .011 | .112 | 9.395 | .000 | .079 | .121 |
| MI | .009 | .014 | .009 | .628 | .530 | -.019 | .037 |
| FH | .039 | .013 | .035 | 3.092 | .002 | .014 | .063 |
| lHDL | .150 | .010 | .167 | 14.936 | .000 | .130 | .170 |
| hChol | .157 | .011 | .171 | 14.130 | .000 | .135 | .179 |
| hLDL | .140 | .015 | .111 | 9.491 | .000 | .111 | .169 |
| CAD | .054 | .013 | .060 | 4.048 | .000 | .028 | .080 |
| Age (Binned) | -.031 | .004 | -.098 | -8.128 | .000 | -.038 | -.023 |
| rs3729855C | .155 | .182 | .065 | .853 | .394 | -.202 | .512 |
| rs3729855T | .229 | .184 | .092 | 1.246 | .213 | -.132 | .590 |
| rs3729856AA | -.754 | .295 | -.646 | -2.552 | .011 | -1.333 | -.175 |
| rs3729856G | -.775 | .296 | -.657 | -2.620 | .009 | -1.354 | -.195 |
| rs1062219C | .225 | .158 | .250 | 1.419 | .156 | -.086 | .535 |
| rs1062219T | .209 | .158 | .233 | 1.323 | .186 | -.101 | .519 |
| rs12825C | -.260 | .087 | -.289 | -2.977 | .003 | -.431 | -.089 |
| rs12825G | -.273 | .087 | -.304 | -3.135 | .002 | -.443 | -.102 |
| ra804291AA | .000 | .129 | .000 | .002 | .998 | -.253 | .254 |
| ra804291G | -.002 | .129 | -.003 | -.019 | .985 | -.255 | .250 |
| rs11785481C | .241 | .179 | .175 | 1.343 | .179 | -.111 | .592 |
| rs11785481T | .229 | .180 | .164 | 1.272 | .203 | -.124 | .582 |
| rs3203358C | .389 | .198 | .351 | 1.964 | .050 | .001 | .777 |
| rs3203358G | .400 | .198 | .358 | 2.016 | .044 | .011 | .789 |
| rs2740434G | .170 | .131 | .168 | 1.299 | .194 | -.086 | .426 |
| rs2740434AA | .169 | .131 | .165 | 1.288 | .198 | -.088 | .425 |
| rs17153743AA | .128 | .128 | .035 | .996 | .319 | -.124 | .379 |
| rs17153743G | .126 | .136 | .031 | .931 | .352 | -.140 | .392 |
| rs13264774C | -.001 | .029 | -.001 | -.032 | .975 | -.058 | .056 |
| rs13264774T | .008 | .032 | .006 | .241 | .809 | -.055 | .070 |
| rs804280AA | -.054 | .098 | -.060 | -.554 | .580 | -.247 | .138 |
| rs804280C | -.029 | .098 | -.032 | -.298 | .766 | -.221 | .163 |

**SNP Interaction with low high density lipoprotein levels**

| Model | Unstandardized Coefficients | | Standardized Coefficients | t | P-Value | 95.0% Confidence Interval for B | |
| --- | --- | --- | --- | --- | --- | --- | --- |
|  | B | Standard Error | Beta |  |  | Lower Bound | Upper Bound |
| (Constant) | .691 | .168 |  | 4.104 | .000 | .361 | 1.022 |
| Sex | -.174 | .012 | -.166 | -14.333 | .000 | -.198 | -.150 |
| hTG | .191 | .013 | .171 | 14.936 | .000 | .166 | .216 |
| OBS | -.003 | .011 | -.003 | -.297 | .767 | -.026 | .019 |
| T2DM | .060 | .012 | .060 | 4.990 | .000 | .037 | .084 |
| MI | .065 | .016 | .061 | 4.056 | .000 | .034 | .097 |
| FH | -.014 | .014 | -.011 | -.994 | .320 | -.042 | .014 |
| HChol | -.059 | .013 | -.057 | -4.621 | .000 | -.083 | -.034 |
| hLDL | -.073 | .017 | -.052 | -4.367 | .000 | -.106 | -.040 |
| CAD | .080 | .015 | .080 | 5.323 | .000 | .051 | .109 |
| Age (Binned) | -.015 | .004 | -.042 | -3.424 | .001 | -.023 | -.006 |
| rs3729855C | .465 | .205 | .176 | 2.266 | .024 | .063 | .868 |
| rs3729855T | .509 | .208 | .183 | 2.452 | .014 | .102 | .917 |
| rs3729856AA | -.159 | .333 | -.122 | -.478 | .633 | -.813 | .494 |
| rs3729856G | -.155 | .334 | -.118 | -.465 | .642 | -.810 | .499 |
| rs1062219C | -.199 | .179 | -.199 | -1.115 | .265 | -.549 | .151 |
| rs1062219T | -.214 | .178 | -.214 | -1.200 | .230 | -.564 | .136 |
| rs12825C | .068 | .099 | .068 | .689 | .491 | -.125 | .261 |
| rs12825G | .072 | .098 | .072 | .731 | .465 | -.121 | .264 |
| ra804291AA | .003 | .146 | .003 | .022 | .982 | -.283 | .289 |
| ra804291G | -.005 | .145 | -.005 | -.032 | .975 | -.289 | .280 |
| rs11785481C | -.002 | .202 | -.001 | -.008 | .994 | -.398 | .395 |
| rs11785481T | -.013 | .203 | -.009 | -.065 | .948 | -.412 | .385 |
| rs3203358C | -.460 | .224 | -.373 | -2.060 | .039 | -.899 | -.022 |
| rs3203358G | -.455 | .224 | -.365 | -2.031 | .042 | -.893 | -.016 |
| rs2740434G | -.020 | .147 | -.018 | -.137 | .891 | -.309 | .269 |
| rs2740434AA | -.041 | .148 | -.036 | -.275 | .784 | -.330 | .249 |
| rs17153743AA | .049 | .145 | .012 | .341 | .733 | -.234 | .333 |
| rs17153743G | .019 | .153 | .004 | .125 | .900 | -.281 | .319 |
| rs13264774C | -.008 | .033 | -.006 | -.246 | .805 | -.072 | .056 |
| rs13264774T | -.017 | .036 | -.012 | -.486 | .627 | -.088 | .053 |
| rs804280AA | .165 | .111 | .165 | 1.493 | .135 | -.052 | .382 |
| rs804280C | .179 | .111 | .179 | 1.624 | .104 | -.037 | .396 |

SNP Interaction with low high density lipoprotein –cholesterol levels

| Model | Unstandardized Coefficients | | Standardized Coefficients | t | P-Value | 95.0% Confidence Interval for B | |
| --- | --- | --- | --- | --- | --- | --- | --- |
|  | B | Standard Error | Beta |  |  | Lower Bound | Upper Bound |
| (Constant) | -.125 | .153 |  | -.818 | .413 | -.424 | .174 |
| Sex | .018 | .011 | .017 | 1.596 | .110 | -.004 | .040 |
| lHDL | -.048 | .010 | -.049 | -4.621 | .000 | -.068 | -.028 |
| hTG | .164 | .012 | .150 | 14.130 | .000 | .141 | .186 |
| OBS | .069 | .010 | .070 | 6.691 | .000 | .049 | .089 |
| T2DM | .071 | .011 | .073 | 6.486 | .000 | .050 | .092 |
| MI | .047 | .015 | .045 | 3.195 | .001 | .018 | .075 |
| FH | .029 | .013 | .024 | 2.232 | .026 | .003 | .054 |
| hLDL | .473 | .014 | .345 | 33.474 | .000 | .445 | .501 |
| CAD | .134 | .014 | .137 | 9.893 | .000 | .107 | .160 |
| Age (Binned) | .016 | .004 | .047 | 4.129 | .000 | .008 | .024 |
| rs3729855C | .313 | .186 | .121 | 1.683 | .093 | -.052 | .677 |
| rs3729855T | .318 | .188 | .117 | 1.691 | .091 | -.051 | .687 |
| rs3729856AA | -.458 | .302 | -.361 | -1.518 | .129 | -1.050 | .133 |
| rs3729856G | -.468 | .302 | -.365 | -1.550 | .121 | -1.061 | .124 |
| rs1062219C | .285 | .162 | .292 | 1.765 | .078 | -.032 | .602 |
| rs1062219T | .308 | .161 | .315 | 1.910 | .056 | -.008 | .625 |
| rs12825C | -.253 | .089 | -.259 | -2.840 | .005 | -.428 | -.078 |
| rs12825G | -.248 | .089 | -.255 | -2.795 | .005 | -.422 | -.074 |
| ra804291AA | -.141 | .132 | -.145 | -1.064 | .287 | -.400 | .118 |
| ra804291G | -.136 | .131 | -.140 | -1.035 | .301 | -.393 | .121 |
| rs11785481C | .095 | .183 | .064 | .520 | .603 | -.264 | .454 |
| rs11785481T | .101 | .184 | .066 | .548 | .584 | -.260 | .462 |
| rs3203358C | -.122 | .202 | -.101 | -.601 | .548 | -.518 | .275 |
| rs3203358G | -.137 | .203 | -.113 | -.675 | .500 | -.534 | .260 |
| rs2740434G | .282 | .133 | .256 | 2.115 | .035 | .021 | .543 |
| rs2740434AA | .291 | .134 | .262 | 2.178 | .029 | .029 | .554 |
| rs17153743AA | .187 | .131 | .047 | 1.427 | .154 | -.070 | .444 |
| rs17153743G | .100 | .138 | .023 | .720 | .471 | -.172 | .371 |
| rs13264774C | -.024 | .030 | -.018 | -.800 | .424 | -.082 | .034 |
| rs13264774T | -.020 | .033 | -.014 | -.618 | .537 | -.084 | .044 |
| rs804280AA | -.012 | .100 | -.012 | -.120 | .904 | -.209 | .184 |
| rs804280C | -.020 | .100 | -.021 | -.203 | .839 | -.216 | .176 |

**SNP Interaction with high low density lipoprotein levels**

| Model | Unstandardized Coefficients | | Standardized Coefficients | t | P-Value | 95.0% Confidence Interval for B | |
| --- | --- | --- | --- | --- | --- | --- | --- |
|  | B | Standard Error | Beta |  |  | Lower Bound | Upper Bound |
| (Constant) | .273 | .116 |  | 2.360 | .018 | .046 | .500 |
| Sex | .010 | .008 | .014 | 1.208 | .227 | -.006 | .027 |
| HChol | .272 | .008 | .374 | 33.474 | .000 | .256 | .288 |
| lHDL | -.034 | .008 | -.048 | -4.367 | .000 | -.050 | -.019 |
| hTG | .084 | .009 | .106 | 9.491 | .000 | .067 | .101 |
| OBS | -.025 | .008 | -.035 | -3.262 | .001 | -.041 | -.010 |
| T2DM | -.012 | .008 | -.017 | -1.480 | .139 | -.029 | .004 |
| MI | -.006 | .011 | -.008 | -.554 | .579 | -.028 | .016 |
| FH | .022 | .010 | .025 | 2.297 | .022 | .003 | .041 |
| CAD | -.025 | .010 | -.036 | -2.453 | .014 | -.046 | -.005 |
| Age (Binned) | -.006 | .003 | -.022 | -1.875 | .061 | -.011 | .000 |
| rs3729855C | -.210 | .141 | -.111 | -1.489 | .136 | -.487 | .066 |
| rs3729855T | -.219 | .143 | -.110 | -1.531 | .126 | -.498 | .061 |
| rs3729856AA | .503 | .229 | .544 | 2.196 | .028 | .054 | .952 |
| rs3729856G | .512 | .229 | .548 | 2.233 | .026 | .063 | .961 |
| rs1062219C | -.134 | .123 | -.188 | -1.090 | .276 | -.374 | .107 |
| rs1062219T | -.144 | .123 | -.201 | -1.171 | .242 | -.384 | .097 |
| rs12825C | .130 | .068 | .183 | 1.920 | .055 | -.003 | .263 |
| rs12825G | .139 | .067 | .196 | 2.065 | .039 | .007 | .271 |
| ra804291AA | .154 | .100 | .217 | 1.534 | .125 | -.043 | .350 |
| ra804291G | .152 | .100 | .215 | 1.524 | .128 | -.044 | .347 |
| rs11785481C | -.139 | .139 | -.127 | -.996 | .319 | -.411 | .134 |
| rs11785481T | -.121 | .140 | -.109 | -.863 | .388 | -.394 | .153 |
| rs3203358C | -.145 | .154 | -.165 | -.946 | .344 | -.447 | .156 |
| rs3203358G | -.137 | .154 | -.155 | -.889 | .374 | -.438 | .165 |
| rs2740434G | -.132 | .101 | -.164 | -1.300 | .194 | -.330 | .067 |
| rs2740434AA | -.130 | .102 | -.161 | -1.281 | .200 | -.329 | .069 |
| rs17153743AA | -.281 | .099 | -.098 | -2.828 | .005 | -.476 | -.086 |
| rs17153743G | -.240 | .105 | -.075 | -2.287 | .022 | -.446 | -.034 |
| rs13264774C | .005 | .022 | .005 | .221 | .825 | -.039 | .049 |
| rs13264774T | .001 | .025 | .001 | .048 | .962 | -.047 | .050 |
| rs804280AA | .041 | .076 | .057 | .536 | .592 | -.108 | .190 |
| rs804280C | .037 | .076 | .052 | .484 | .628 | -.112 | .186 |

**Myocardial Infarction interaction with age**

| Parameter | B | Std. Error | t | Sig. | 95% Confidence Interval | |
| --- | --- | --- | --- | --- | --- | --- |
|  |  |  |  |  | Lower Bound | Upper Bound |
| Intercept | .531 | .254 | 2.088 | .037 | .032 | 1.029 |
| [Age_New=1] * rs3729855C | .700 | .358 | 1.955 | .051 | -.002 | 1.402 |
| [Age_New=2] * rs3729855C | -.383 | .621 | -.616 | .538 | -1.599 | .834 |
| [Age_New=3] * rs3729855C | .347 | .324 | 1.072 | .284 | -.288 | .983 |
| [Age_New=4] * rs3729855C | -.121 | .317 | -.382 | .703 | -.741 | .500 |
| [Age_New=5] * rs3729855C | .465 | .406 | 1.147 | .251 | -.330 | 1.260 |
| [Age_New=1] * rs3729855T | .625 | .363 | 1.723 | .085 | -.086 | 1.335 |
| [Age_New=2] * rs3729855T | -.571 | .619 | -.922 | .357 | -1.785 | .643 |
| [Age_New=3] * rs3729855T | .448 | .329 | 1.361 | .174 | -.197 | 1.092 |
| [Age_New=4] * rs3729855T | -.183 | .324 | -.565 | .572 | -.817 | .451 |
| [Age_New=5] * rs3729855T | .387 | .412 | .939 | .348 | -.420 | 1.193 |
| [Age_New=1] * rs3729856AA | -.881 | .603 | -1.460 | .144 | -2.063 | .301 |
| [Age_New=2] * rs3729856AA | -.036 | .376 | -.095 | .924 | -.773 | .702 |
| [Age_New=3] * rs3729856AA | -.436 | .290 | -1.507 | .132 | -1.004 | .131 |
| [Age_New=4] * rs3729856AA | -.029 | .034 | -.854 | .393 | -.097 | .038 |
| [Age_New=5] * rs3729856AA | .026 | .034 | .758 | .448 | -.041 | .094 |
| [Age_New=1] * rs3729856G | -.825 | .603 | -1.368 | .171 | -2.007 | .357 |
| [Age_New=2] * rs3729856G | -.060 | .377 | -.159 | .873 | -.799 | .679 |
| [Age_New=3] * rs3729856G | -.401 | .291 | -1.377 | .169 | -.972 | .170 |
| [Age_New=4] * rs3729856G | 0^a^ |  |  |  |  |  |
| [Age_New=5] * rs3729856G | 0^a^ |  |  |  |  |  |
| [Age_New=1] * rs1062219C | .112 | .320 | .349 | .727 | -.516 | .740 |
| [Age_New=2] * rs1062219C | .059 | .027 | 2.145 | .032 | .005 | .113 |
| [Age_New=3] * rs1062219C | -.001 | .028 | -.019 | .985 | -.056 | .055 |
| [Age_New=4] * rs1062219C | -.002 | .429 | -.005 | .996 | -.843 | .839 |
| [Age_New=5] * rs1062219C | .016 | .029 | .535 | .593 | -.041 | .073 |
| [Age_New=1] * rs1062219T | .129 | .320 | .403 | .687 | -.499 | .757 |
| [Age_New=2] * rs1062219T | 0^a^ |  |  |  |  |  |
| [Age_New=3] * rs1062219T | 0^a^ |  |  |  |  |  |
| [Age_New=4] * rs1062219T | .002 | .429 | .005 | .996 | -.839 | .843 |
| [Age_New=5] * rs1062219T | 0^a^ |  |  |  |  |  |
| [Age_New=1] * rs12825C | -.362 | .236 | -1.536 | .125 | -.824 | .100 |
| [Age_New=2] * rs12825C | .167 | .217 | .770 | .441 | -.258 | .591 |
| [Age_New=3] * rs12825C | -.050 | .133 | -.377 | .706 | -.312 | .211 |
| [Age_New=4] * rs12825C | .038 | .028 | 1.369 | .171 | -.016 | .092 |
| [Age_New=5] * rs12825C | -.157 | .215 | -.730 | .465 | -.579 | .265 |
| [Age_New=1] * rs12825G | -.351 | .235 | -1.496 | .135 | -.811 | .109 |
| [Age_New=2] * rs12825G | .200 | .216 | .928 | .353 | -.223 | .623 |
| [Age_New=3] * rs12825G | -.075 | .133 | -.565 | .572 | -.335 | .185 |
| [Age_New=4] * rs12825G | 0^a^ |  |  |  |  |  |
| [Age_New=5] * rs12825G | -.144 | .216 | -.670 | .503 | -.567 | .278 |
| [Age_New=1] * rs11785481C | -.009 | .290 | -.030 | .976 | -.578 | .560 |
| [Age_New=2] * rs11785481C | -.176 | .434 | -.405 | .686 | -1.026 | .675 |
| [Age_New=3] * rs11785481C | .006 | .035 | .183 | .855 | -.063 | .076 |
| [Age_New=4] * rs11785481C | -.011 | .041 | -.261 | .794 | -.091 | .070 |
| [Age_New=5] * rs11785481C | .006 | .038 | .151 | .880 | -.069 | .080 |
| [Age_New=1] * rs11785481T | -.048 | .292 | -.166 | .868 | -.621 | .524 |
| [Age_New=2] * rs11785481T | -.189 | .436 | -.433 | .665 | -1.043 | .665 |
| [Age_New=3] * rs11785481T | 0^a^ |  |  |  |  |  |
| [Age_New=4] * rs11785481T | 0^a^ |  |  |  |  |  |
| [Age_New=5] * rs11785481T | 0^a^ |  |  |  |  |  |
| [Age_New=1] * rs3203358C | .137 | .404 | .340 | .734 | -.654 | .928 |
| [Age_New=2] * rs3203358C | .633 | .589 | 1.075 | .283 | -.522 | 1.789 |
| [Age_New=3] * rs3203358C | .005 | .029 | .178 | .859 | -.052 | .063 |
| [Age_New=4] * rs3203358C | -.045 | .030 | -1.496 | .135 | -.104 | .014 |
| [Age_New=5] * rs3203358C | .021 | .031 | .672 | .501 | -.040 | .081 |
| [Age_New=1] * rs3203358G | .080 | .403 | .199 | .842 | -.710 | .870 |
| [Age_New=2] * rs3203358G | .614 | .591 | 1.040 | .299 | -.544 | 1.772 |
| [Age_New=3] * rs3203358G | 0^a^ |  |  |  |  |  |
| [Age_New=4] * rs3203358G | 0^a^ |  |  |  |  |  |
| [Age_New=5] * rs3203358G | 0^a^ |  |  |  |  |  |
| [Age_New=1] * rs2740434G | .452 | .387 | 1.169 | .242 | -.306 | 1.210 |
| [Age_New=2] * rs2740434G | .003 | .429 | .008 | .994 | -.837 | .844 |
| [Age_New=3] * rs2740434G | -.007 | .228 | -.032 | .975 | -.454 | .439 |
| [Age_New=4] * rs2740434G | -.035 | .029 | -1.232 | .218 | -.092 | .021 |
| [Age_New=5] * rs2740434G | -.064 | .434 | -.147 | .883 | -.915 | .788 |
| [Age_New=1] * rs2740434AA | .444 | .388 | 1.143 | .253 | -.317 | 1.204 |
| [Age_New=2] * rs2740434AA | .025 | .430 | .057 | .954 | -.818 | .868 |
| [Age_New=3] * rs2740434AA | -.037 | .228 | -.162 | .872 | -.484 | .410 |
| [Age_New=4] * rs2740434AA | 0^a^ |  |  |  |  |  |
| [Age_New=5] * rs2740434AA | -.087 | .435 | -.199 | .842 | -.940 | .767 |
| [Age_New=1] * rs17153743AA | .240 | .247 | .974 | .330 | -.243 | .724 |
| [Age_New=2] * rs17153743AA | .028 | .078 | .365 | .715 | -.124 | .181 |
| [Age_New=3] * rs17153743AA | .344 | .314 | 1.094 | .274 | -.272 | .959 |
| [Age_New=4] * rs17153743AA | .469 | .396 | 1.187 | .235 | -.306 | 1.245 |
| [Age_New=5] * rs17153743AA | -.301 | .431 | -.698 | .485 | -1.145 | .544 |
| [Age_New=1] * rs17153743G | .289 | .263 | 1.099 | .272 | -.227 | .806 |
| [Age_New=2] * rs17153743G | 0^a^ |  |  |  |  |  |
| [Age_New=3] * rs17153743G | .293 | .328 | .895 | .371 | -.349 | .935 |
| [Age_New=4] * rs17153743G | .289 | .410 | .705 | .481 | -.515 | 1.094 |
| [Age_New=5] * rs17153743G | -.251 | .440 | -.569 | .569 | -1.113 | .612 |
| [Age_New=1] * rs13264774C | .102 | .062 | 1.647 | .100 | -.019 | .223 |
| [Age_New=2] * rs13264774C | .172 | .058 | 2.963 | .003 | .058 | .285 |
| [Age_New=3] * rs13264774C | .027 | .058 | .462 | .644 | -.087 | .141 |
| [Age_New=4] * rs13264774C | .003 | .071 | .047 | .962 | -.136 | .142 |
| [Age_New=5] * rs13264774C | -.043 | .067 | -.645 | .519 | -.174 | .088 |
| [Age_New=1] * rs13264774T | .062 | .068 | .911 | .362 | -.071 | .194 |
| [Age_New=2] * rs13264774T | .173 | .065 | 2.674 | .008 | .046 | .301 |
| [Age_New=3] * rs13264774T | .050 | .065 | .769 | .442 | -.078 | .178 |
| [Age_New=4] * rs13264774T | .031 | .077 | .400 | .689 | -.120 | .181 |
| [Age_New=5] * rs13264774T | -.083 | .073 | -1.148 | .251 | -.226 | .059 |
| [Age_New=1] * rs804280AA | -.685 | .304 | -2.254 | .024 | -1.282 | -.089 |
| [Age_New=2] * rs804280AA | -.337 | .304 | -1.110 | .267 | -.934 | .259 |
| [Age_New=3] * rs804280AA | .362 | .161 | 2.253 | .024 | .047 | .677 |
| [Age_New=4] * rs804280AA | -.006 | .031 | -.193 | .847 | -.066 | .054 |
| [Age_New=5] * rs804280AA | .372 | .217 | 1.709 | .087 | -.055 | .798 |
| [Age_New=1] * rs804280C | -.676 | .304 | -2.222 | .026 | -1.272 | -.080 |
| [Age_New=2] * rs804280C | -.240 | .304 | -.790 | .430 | -.837 | .356 |
| [Age_New=3] * rs804280C | .425 | .160 | 2.663 | .008 | .112 | .738 |
| [Age_New=4] * rs804280C | 0^a^ |  |  |  |  |  |
| [Age_New=5] * rs804280C | .365 | .218 | 1.672 | .095 | -.063 | .792 |
| [Age_New=1] * rs804291AA | -.015 | .347 | -.043 | .965 | -.696 | .665 |
| [Age_New=2] * rs804291AA | -.007 | .031 | -.224 | .823 | -.067 | .053 |
| [Age_New=3] * rs804291AA | -.365 | .287 | -1.269 | .204 | -.928 | .199 |
| [Age_New=4] * rs804291AA | .005 | .034 | .137 | .891 | -.062 | .071 |
| [Age_New=5] * rs804291AA | -.022 | .033 | -.651 | .515 | -.086 | .043 |
| [Age_New=1] * rs804291G | -.019 | .346 | -.054 | .957 | -.697 | .660 |
| [Age_New=2] * rs804291G | 0^a^ |  |  |  |  |  |
| [Age_New=3] * rs804291G | -.407 | .286 | -1.422 | .155 | -.969 | .154 |
| [Age_New=4] * rs804291G | 0^a^ |  |  |  |  |  |
| [Age_New=5] * rs804291G | 0^a^ |  |  |  |  |  |
|  | | | | | | |

**TYPE 2 DIABETES MELLITUS Interaction with age**

| Parameter | B | Std. Error | t | Sig. | 95% Confidence Interval | |
| --- | --- | --- | --- | --- | --- | --- |
|  |  |  |  |  | Lower Bound | Upper Bound |
| Intercept | .655 | .275 | 2.382 | .017 | .116 | 1.194 |
| [Age_New=1] * rs3729855C | -.637 | .387 | -1.645 | .100 | -1.397 | .122 |
| [Age_New=2] * rs3729855C | .477 | .672 | .709 | .478 | -.840 | 1.793 |
| [Age_New=3] * rs3729855C | -.287 | .351 | -.817 | .414 | -.974 | .401 |
| [Age_New=4] * rs3729855C | .759 | .343 | 2.214 | .027 | .087 | 1.430 |
| [Age_New=5] * rs3729855C | .284 | .439 | .647 | .518 | -.576 | 1.144 |
| [Age_New=1] * rs3729855T | -.760 | .392 | -1.938 | .053 | -1.529 | .009 |
| [Age_New=2] * rs3729855T | .455 | .670 | .678 | .497 | -.859 | 1.769 |
| [Age_New=3] * rs3729855T | -.332 | .356 | -.932 | .351 | -1.029 | .366 |
| [Age_New=4] * rs3729855T | .598 | .350 | 1.709 | .087 | -.088 | 1.285 |
| [Age_New=5] * rs3729855T | .190 | .445 | .427 | .670 | -.683 | 1.063 |
| [Age_New=1] * rs3729856AA | -.367 | .653 | -.563 | .573 | -1.647 | .912 |
| [Age_New=2] * rs3729856AA | -.007 | .407 | -.018 | .986 | -.805 | .791 |
| [Age_New=3] * rs3729856AA | .745 | .313 | 2.376 | .018 | .130 | 1.359 |
| [Age_New=4] * rs3729856AA | -.010 | .037 | -.264 | .791 | -.083 | .063 |
| [Age_New=5] * rs3729856AA | .007 | .037 | .188 | .851 | -.066 | .080 |
| [Age_New=1] * rs3729856G | -.343 | .653 | -.525 | .600 | -1.622 | .937 |
| [Age_New=2] * rs3729856G | -.030 | .408 | -.074 | .941 | -.830 | .769 |
| [Age_New=3] * rs3729856G | .756 | .315 | 2.398 | .016 | .138 | 1.374 |
| [Age_New=4] * rs3729856G | 0^a^ |  |  |  |  |  |
| [Age_New=5] * rs3729856G | 0^a^ |  |  |  |  |  |
| [Age_New=1] * rs1062219C | -.137 | .347 | -.395 | .693 | -.816 | .543 |
| [Age_New=2] * rs1062219C | .017 | .030 | .568 | .570 | -.041 | .075 |
| [Age_New=3] * rs1062219C | .028 | .031 | .905 | .365 | -.032 | .088 |
| [Age_New=4] * rs1062219C | .000 | .464 | .001 | .999 | -.909 | .910 |
| [Age_New=5] * rs1062219C | -.001 | .031 | -.037 | .971 | -.063 | .060 |
| [Age_New=1] * rs1062219T | -.127 | .347 | -.368 | .713 | -.807 | .552 |
| [Age_New=2] * rs1062219T | 0^a^ |  |  |  |  |  |
| [Age_New=3] * rs1062219T | 0^a^ |  |  |  |  |  |
| [Age_New=4] * rs1062219T | .000 | .464 | -.001 | .999 | -.910 | .909 |
| [Age_New=5] * rs1062219T | 0^a^ |  |  |  |  |  |
| [Age_New=1] * rs12825C | .116 | .255 | .455 | .649 | -.384 | .616 |
| [Age_New=2] * rs12825C | -.420 | .234 | -1.792 | .073 | -.879 | .039 |
| [Age_New=3] * rs12825C | .505 | .144 | 3.501 | .000 | .222 | .788 |
| [Age_New=4] * rs12825C | -.020 | .030 | -.677 | .498 | -.078 | .038 |
| [Age_New=5] * rs12825C | -.349 | .233 | -1.496 | .135 | -.805 | .108 |
| [Age_New=1] * rs12825G | .114 | .254 | .449 | .653 | -.384 | .612 |
| [Age_New=2] * rs12825G | -.429 | .234 | -1.838 | .066 | -.887 | .029 |
| [Age_New=3] * rs12825G | .480 | .143 | 3.349 | .001 | .199 | .761 |
| [Age_New=4] * rs12825G | 0^a^ |  |  |  |  |  |
| [Age_New=5] * rs12825G | -.334 | .233 | -1.433 | .152 | -.792 | .123 |
| [Age_New=1] * rs11785481C | -.018 | .314 | -.057 | .954 | -.634 | .598 |
| [Age_New=2] * rs11785481C | .966 | .469 | 2.059 | .040 | .046 | 1.886 |
| [Age_New=3] * rs11785481C | .016 | .038 | .425 | .671 | -.059 | .091 |
| [Age_New=4] * rs11785481C | .003 | .044 | .074 | .941 | -.084 | .090 |
| [Age_New=5] * rs11785481C | -.053 | .041 | -1.293 | .196 | -.133 | .027 |
| [Age_New=1] * rs11785481T | -.098 | .316 | -.310 | .756 | -.718 | .522 |
| [Age_New=2] * rs11785481T | 1.055 | .471 | 2.238 | .025 | .131 | 1.979 |
| [Age_New=3] * rs11785481T | 0^a^ |  |  |  |  |  |
| [Age_New=4] * rs11785481T | 0^a^ |  |  |  |  |  |
| [Age_New=5] * rs11785481T | 0^a^ |  |  |  |  |  |
| [Age_New=1] * rs3203358C | .833 | .437 | 1.907 | .057 | -.023 | 1.689 |
| [Age_New=2] * rs3203358C | -.679 | .638 | -1.065 | .287 | -1.929 | .571 |
| [Age_New=3] * rs3203358C | -.016 | .032 | -.510 | .610 | -.078 | .046 |
| [Age_New=4] * rs3203358C | -.027 | .033 | -.814 | .416 | -.091 | .038 |
| [Age_New=5] * rs3203358C | .010 | .033 | .290 | .772 | -.056 | .075 |
| [Age_New=1] * rs3203358G | .776 | .436 | 1.781 | .075 | -.078 | 1.631 |
| [Age_New=2] * rs3203358G | -.673 | .639 | -1.054 | .292 | -1.926 | .579 |
| [Age_New=3] * rs3203358G | 0^a^ |  |  |  |  |  |
| [Age_New=4] * rs3203358G | 0^a^ |  |  |  |  |  |
| [Age_New=5] * rs3203358G | 0^a^ |  |  |  |  |  |
| [Age_New=1] * rs2740434G | .635 | .419 | 1.516 | .130 | -.186 | 1.455 |
| [Age_New=2] * rs2740434G | -.008 | .464 | -.018 | .986 | -.918 | .901 |
| [Age_New=3] * rs2740434G | -.381 | .246 | -1.545 | .122 | -.864 | .102 |
| [Age_New=4] * rs2740434G | .003 | .031 | .109 | .913 | -.058 | .064 |
| [Age_New=5] * rs2740434G | -.901 | .470 | -1.918 | .055 | -1.823 | .020 |
| [Age_New=1] * rs2740434AA | .646 | .420 | 1.538 | .124 | -.177 | 1.469 |
| [Age_New=2] * rs2740434AA | -.031 | .465 | -.067 | .947 | -.943 | .881 |
| [Age_New=3] * rs2740434AA | -.343 | .247 | -1.390 | .165 | -.827 | .141 |
| [Age_New=4] * rs2740434AA | 0^a^ |  |  |  |  |  |
| [Age_New=5] * rs2740434AA | -.934 | .471 | -1.982 | .047 | -1.858 | -.010 |
| [Age_New=1] * rs17153743AA | -.328 | .267 | -1.227 | .220 | -.851 | .196 |
| [Age_New=2] * rs17153743AA | -.043 | .084 | -.505 | .613 | -.208 | .123 |
| [Age_New=3] * rs17153743AA | -.081 | .340 | -.238 | .812 | -.747 | .585 |
| [Age_New=4] * rs17153743AA | -.655 | .428 | -1.531 | .126 | -1.494 | .184 |
| [Age_New=5] * rs17153743AA | .275 | .466 | .591 | .554 | -.638 | 1.189 |
| [Age_New=1] * rs17153743G | -.219 | .285 | -.769 | .442 | -.778 | .339 |
| [Age_New=2] * rs17153743G | 0^a^ |  |  |  |  |  |
| [Age_New=3] * rs17153743G | -.165 | .354 | -.466 | .641 | -.860 | .530 |
| [Age_New=4] * rs17153743G | -.658 | .444 | -1.482 | .138 | -1.529 | .212 |
| [Age_New=5] * rs17153743G | .427 | .476 | .897 | .370 | -.506 | 1.361 |
| [Age_New=1] * rs13264774C | .093 | .067 | 1.391 | .164 | -.038 | .224 |
| [Age_New=2] * rs13264774C | .051 | .063 | .821 | .412 | -.071 | .174 |
| [Age_New=3] * rs13264774C | .168 | .063 | 2.657 | .008 | .044 | .292 |
| [Age_New=4] * rs13264774C | -.059 | .077 | -.773 | .439 | -.210 | .091 |
| [Age_New=5] * rs13264774C | .102 | .072 | 1.406 | .160 | -.040 | .243 |
| [Age_New=1] * rs13264774T | .064 | .073 | .872 | .383 | -.080 | .207 |
| [Age_New=2] * rs13264774T | .044 | .070 | .625 | .532 | -.094 | .181 |
| [Age_New=3] * rs13264774T | .151 | .070 | 2.145 | .032 | .013 | .289 |
| [Age_New=4] * rs13264774T | -.024 | .083 | -.294 | .769 | -.187 | .138 |
| [Age_New=5] * rs13264774T | .028 | .079 | .353 | .724 | -.126 | .182 |
| [Age_New=1] * rs804280AA | -.792 | .329 | -2.407 | .016 | -1.437 | -.147 |
| [Age_New=2] * rs804280AA | -.466 | .329 | -1.417 | .157 | -1.111 | .179 |
| [Age_New=3] * rs804280AA | -.448 | .174 | -2.576 | .010 | -.789 | -.107 |
| [Age_New=4] * rs804280AA | .021 | .033 | .622 | .534 | -.044 | .086 |
| [Age_New=5] * rs804280AA | .620 | .235 | 2.636 | .008 | .159 | 1.081 |
| [Age_New=1] * rs804280C | -.799 | .329 | -2.429 | .015 | -1.445 | -.154 |
| [Age_New=2] * rs804280C | -.436 | .329 | -1.325 | .185 | -1.082 | .209 |
| [Age_New=3] * rs804280C | -.371 | .173 | -2.147 | .032 | -.710 | -.032 |
| [Age_New=4] * rs804280C | 0^a^ |  |  |  |  |  |
| [Age_New=5] * rs804280C | .647 | .236 | 2.741 | .006 | .184 | 1.109 |
| [Age_New=1] * rs804291AA | .172 | .376 | .457 | .647 | -.564 | .908 |
| [Age_New=2] * rs804291AA | .017 | .033 | .499 | .618 | -.048 | .082 |
| [Age_New=3] * rs804291AA | -.232 | .311 | -.747 | .455 | -.842 | .377 |
| [Age_New=4] * rs804291AA | .007 | .037 | .200 | .842 | -.065 | .079 |
| [Age_New=5] * rs804291AA | .025 | .036 | .687 | .492 | -.046 | .095 |
| [Age_New=1] * rs804291G | .212 | .375 | .565 | .572 | -.523 | .946 |
| [Age_New=2] * rs804291G | 0^a^ |  |  |  |  |  |
| [Age_New=3] * rs804291G | -.236 | .310 | -.761 | .447 | -.843 | .372 |
| [Age_New=4] * rs804291G | 0^a^ |  |  |  |  |  |
| [Age_New=5] * rs804291G | 0^a^ |  |  |  |  |  |

**HYPERTENSION** INTERACTION WITH AGE

| Parameter | B | Std. Error | t | Sig. | 95% Confidence Interval | |
| --- | --- | --- | --- | --- | --- | --- |
|  |  |  |  |  | Lower Bound | Upper Bound |
| Intercept | .925 | .233 | 3.972 | .000 | .469 | 1.382 |
| [Age_New=1] * rs3729855C | -.272 | .328 | -.830 | .407 | -.916 | .371 |
| [Age_New=2] * rs3729855C | .064 | .569 | .112 | .911 | -1.052 | 1.179 |
| [Age_New=3] * rs3729855C | .558 | .297 | 1.879 | .060 | -.024 | 1.141 |
| [Age_New=4] * rs3729855C | .146 | .290 | .502 | .616 | -.423 | .715 |
| [Age_New=5] * rs3729855C | .028 | .372 | .074 | .941 | -.701 | .756 |
| [Age_New=1] * rs3729855T | -.080 | .332 | -.240 | .810 | -.731 | .572 |
| [Age_New=2] * rs3729855T | -.005 | .568 | -.009 | .993 | -1.118 | 1.108 |
| [Age_New=3] * rs3729855T | .560 | .301 | 1.859 | .063 | -.031 | 1.151 |
| [Age_New=4] * rs3729855T | .229 | .297 | .772 | .440 | -.352 | .810 |
| [Age_New=5] * rs3729855T | .053 | .377 | .140 | .889 | -.687 | .792 |
| [Age_New=1] * rs3729856AA | .014 | .553 | .026 | .979 | -1.070 | 1.098 |
| [Age_New=2] * rs3729856AA | .432 | .345 | 1.253 | .210 | -.244 | 1.108 |
| [Age_New=3] * rs3729856AA | .273 | .266 | 1.027 | .304 | -.248 | .793 |
| [Age_New=4] * rs3729856AA | -.059 | .032 | -1.869 | .062 | -.121 | .003 |
| [Age_New=5] * rs3729856AA | -.040 | .032 | -1.254 | .210 | -.102 | .022 |
| [Age_New=1] * rs3729856G | .047 | .553 | .085 | .932 | -1.037 | 1.131 |
| [Age_New=2] * rs3729856G | .450 | .346 | 1.302 | .193 | -.228 | 1.127 |
| [Age_New=3] * rs3729856G | .273 | .267 | 1.022 | .307 | -.251 | .796 |
| [Age_New=4] * rs3729856G | 0^a^ |  |  |  |  |  |
| [Age_New=5] * rs3729856G | 0^a^ |  |  |  |  |  |
| [Age_New=1] * rs1062219C | .175 | .294 | .597 | .550 | -.400 | .751 |
| [Age_New=2] * rs1062219C | .005 | .025 | .210 | .834 | -.044 | .055 |
| [Age_New=3] * rs1062219C | -.023 | .026 | -.903 | .367 | -.074 | .027 |
| [Age_New=4] * rs1062219C | .004 | .393 | .010 | .992 | -.767 | .775 |
| [Age_New=5] * rs1062219C | -.016 | .027 | -.588 | .557 | -.068 | .037 |
| [Age_New=1] * rs1062219T | .137 | .294 | .468 | .640 | -.438 | .713 |
| [Age_New=2] * rs1062219T | 0^a^ |  |  |  |  |  |
| [Age_New=3] * rs1062219T | 0^a^ |  |  |  |  |  |
| [Age_New=4] * rs1062219T | -.004 | .393 | -.010 | .992 | -.775 | .767 |
| [Age_New=5] * rs1062219T | 0^a^ |  |  |  |  |  |
| [Age_New=1] * rs12825C | -.793 | .216 | -3.670 | .000 | -1.216 | -.369 |
| [Age_New=2] * rs12825C | -.225 | .199 | -1.131 | .258 | -.614 | .165 |
| [Age_New=3] * rs12825C | -.216 | .122 | -1.767 | .077 | -.455 | .024 |
| [Age_New=4] * rs12825C | .025 | .025 | .985 | .325 | -.025 | .074 |
| [Age_New=5] * rs12825C | .377 | .197 | 1.912 | .056 | -.010 | .764 |
| [Age_New=1] * rs12825G | -.809 | .215 | -3.760 | .000 | -1.231 | -.387 |
| [Age_New=2] * rs12825G | -.227 | .198 | -1.146 | .252 | -.614 | .161 |
| [Age_New=3] * rs12825G | -.226 | .121 | -1.861 | .063 | -.464 | .012 |
| [Age_New=4] * rs12825G | 0^a^ |  |  |  |  |  |
| [Age_New=5] * rs12825G | .395 | .198 | 1.999 | .046 | .008 | .783 |
| [Age_New=1] * rs11785481C | -.703 | .266 | -2.641 | .008 | -1.224 | -.181 |
| [Age_New=2] * rs11785481C | .046 | .397 | .115 | .909 | -.733 | .825 |
| [Age_New=3] * rs11785481C | .029 | .032 | .904 | .366 | -.034 | .093 |
| [Age_New=4] * rs11785481C | -.086 | .038 | -2.297 | .022 | -.160 | -.013 |
| [Age_New=5] * rs11785481C | .014 | .035 | .412 | .680 | -.054 | .082 |
| [Age_New=1] * rs11785481T | -.734 | .268 | -2.740 | .006 | -1.259 | -.209 |
| [Age_New=2] * rs11785481T | .039 | .399 | .099 | .921 | -.743 | .822 |
| [Age_New=3] * rs11785481T | 0^a^ |  |  |  |  |  |
| [Age_New=4] * rs11785481T | 0^a^ |  |  |  |  |  |
| [Age_New=5] * rs11785481T | 0^a^ |  |  |  |  |  |
| [Age_New=1] * rs3203358C | .432 | .370 | 1.167 | .243 | -.293 | 1.157 |
| [Age_New=2] * rs3203358C | -.005 | .540 | -.010 | .992 | -1.064 | 1.054 |
| [Age_New=3] * rs3203358C | -.025 | .027 | -.917 | .359 | -.077 | .028 |
| [Age_New=4] * rs3203358C | -.015 | .028 | -.528 | .597 | -.069 | .040 |
| [Age_New=5] * rs3203358C | -.018 | .028 | -.627 | .531 | -.073 | .038 |
| [Age_New=1] * rs3203358G | .450 | .369 | 1.217 | .223 | -.274 | 1.174 |
| [Age_New=2] * rs3203358G | -.040 | .541 | -.075 | .940 | -1.102 | 1.021 |
| [Age_New=3] * rs3203358G | 0^a^ |  |  |  |  |  |
| [Age_New=4] * rs3203358G | 0^a^ |  |  |  |  |  |
| [Age_New=5] * rs3203358G | 0^a^ |  |  |  |  |  |
| [Age_New=1] * rs2740434G | .660 | .355 | 1.861 | .063 | -.035 | 1.355 |
| [Age_New=2] * rs2740434G | .012 | .393 | .031 | .976 | -.759 | .783 |
| [Age_New=3] * rs2740434G | .550 | .209 | 2.632 | .008 | .140 | .959 |
| [Age_New=4] * rs2740434G | -.053 | .026 | -2.020 | .043 | -.105 | -.002 |
| [Age_New=5] * rs2740434G | .106 | .398 | .266 | .790 | -.675 | .886 |
| [Age_New=1] * rs2740434AA | .685 | .356 | 1.926 | .054 | -.012 | 1.382 |
| [Age_New=2] * rs2740434AA | .041 | .394 | .103 | .918 | -.732 | .813 |
| [Age_New=3] * rs2740434AA | .559 | .209 | 2.675 | .007 | .149 | .969 |
| [Age_New=4] * rs2740434AA | 0^a^ |  |  |  |  |  |
| [Age_New=5] * rs2740434AA | .088 | .399 | .222 | .825 | -.694 | .871 |
| [Age_New=1] * rs17153743AA | -.522 | .226 | -2.310 | .021 | -.965 | -.079 |
| [Age_New=2] * rs17153743AA | -.196 | .071 | -2.750 | .006 | -.336 | -.056 |
| [Age_New=3] * rs17153743AA | -.530 | .288 | -1.842 | .065 | -1.094 | .034 |
| [Age_New=4] * rs17153743AA | .075 | .363 | .206 | .837 | -.636 | .785 |
| [Age_New=5] * rs17153743AA | -.942 | .395 | -2.387 | .017 | -1.716 | -.168 |
| [Age_New=1] * rs17153743G | -.313 | .241 | -1.296 | .195 | -.786 | .160 |
| [Age_New=2] * rs17153743G | 0^a^ |  |  |  |  |  |
| [Age_New=3] * rs17153743G | -.543 | .300 | -1.809 | .070 | -1.132 | .045 |
| [Age_New=4] * rs17153743G | -.015 | .376 | -.039 | .969 | -.752 | .723 |
| [Age_New=5] * rs17153743G | -.873 | .403 | -2.164 | .031 | -1.664 | -.082 |
| [Age_New=1] * rs13264774C | .010 | .057 | .174 | .862 | -.101 | .121 |
| [Age_New=2] * rs13264774C | -.041 | .053 | -.770 | .442 | -.145 | .063 |
| [Age_New=3] * rs13264774C | .048 | .054 | .905 | .366 | -.056 | .153 |
| [Age_New=4] * rs13264774C | -.126 | .065 | -1.939 | .052 | -.254 | .001 |
| [Age_New=5] * rs13264774C | .078 | .061 | 1.278 | .201 | -.042 | .198 |
| [Age_New=1] * rs13264774T | -.010 | .062 | -.168 | .866 | -.132 | .111 |
| [Age_New=2] * rs13264774T | -.054 | .059 | -.907 | .365 | -.170 | .063 |
| [Age_New=3] * rs13264774T | .046 | .060 | .771 | .441 | -.071 | .163 |
| [Age_New=4] * rs13264774T | -.068 | .070 | -.966 | .334 | -.206 | .070 |
| [Age_New=5] * rs13264774T | .001 | .067 | .011 | .992 | -.130 | .131 |
| [Age_New=1] * rs804280AA | -.495 | .279 | -1.775 | .076 | -1.041 | .052 |
| [Age_New=2] * rs804280AA | -.276 | .279 | -.990 | .322 | -.822 | .270 |
| [Age_New=3] * rs804280AA | -.020 | .147 | -.138 | .890 | -.309 | .268 |
| [Age_New=4] * rs804280AA | .047 | .028 | 1.664 | .096 | -.008 | .102 |
| [Age_New=5] * rs804280AA | .351 | .199 | 1.762 | .078 | -.040 | .742 |
| [Age_New=1] * rs804280C | -.531 | .279 | -1.906 | .057 | -1.078 | .015 |
| [Age_New=2] * rs804280C | -.266 | .279 | -.953 | .341 | -.812 | .281 |
| [Age_New=3] * rs804280C | -.008 | .146 | -.055 | .956 | -.295 | .279 |
| [Age_New=4] * rs804280C | 0^a^ |  |  |  |  |  |
| [Age_New=5] * rs804280C | .356 | .200 | 1.783 | .075 | -.035 | .748 |
| [Age_New=1] * rs804291AA | 1.101 | .318 | 3.460 | .001 | .477 | 1.725 |
| [Age_New=2] * rs804291AA | -.024 | .028 | -.854 | .393 | -.079 | .031 |
| [Age_New=3] * rs804291AA | -.727 | .263 | -2.761 | .006 | -1.244 | -.211 |
| [Age_New=4] * rs804291AA | -.007 | .031 | -.237 | .812 | -.068 | .054 |
| [Age_New=5] * rs804291AA | .033 | .030 | 1.082 | .279 | -.027 | .092 |
| [Age_New=1] * rs804291G | 1.132 | .317 | 3.567 | .000 | .510 | 1.754 |
| [Age_New=2] * rs804291G | 0^a^ |  |  |  |  |  |
| [Age_New=3] * rs804291G | -.734 | .263 | -2.797 | .005 | -1.249 | -.220 |
| [Age_New=4] * rs804291G | 0^a^ |  |  |  |  |  |
| [Age_New=5] * rs804291G | 0^a^ |  |  |  |  |  |

**OBESITY** INTERACTION WITH AGE

| Parameter | B | Std. Error | t | Sig. | 95% Confidence Interval | |
| --- | --- | --- | --- | --- | --- | --- |
|  |  |  |  |  | Lower Bound | Upper Bound |
| Intercept | .138 | .290 | .476 | .634 | -.430 | .705 |
| [Age_New=1] * rs3729855C | -.544 | .408 | -1.333 | .183 | -1.343 | .256 |
| [Age_New=2] * rs3729855C | .143 | .753 | .190 | .849 | -1.333 | 1.619 |
| [Age_New=3] * rs3729855C | .301 | .369 | .814 | .416 | -.423 | 1.025 |
| [Age_New=4] * rs3729855C | -.370 | .362 | -1.020 | .308 | -1.080 | .340 |
| [Age_New=5] * rs3729855C | -.031 | .463 | -.067 | .946 | -.939 | .876 |
| [Age_New=1] * rs3729855T | -.518 | .413 | -1.253 | .210 | -1.328 | .292 |
| [Age_New=2] * rs3729855T | .061 | .755 | .081 | .935 | -1.418 | 1.541 |
| [Age_New=3] * rs3729855T | .197 | .375 | .524 | .600 | -.539 | .933 |
| [Age_New=4] * rs3729855T | -.246 | .370 | -.664 | .507 | -.972 | .480 |
| [Age_New=5] * rs3729855T | -.020 | .470 | -.041 | .967 | -.942 | .903 |
| [Age_New=1] * rs3729856AA | .974 | .687 | 1.417 | .156 | -.373 | 2.320 |
| [Age_New=2] * rs3729856AA | 1.011 | .495 | 2.040 | .041 | .039 | 1.982 |
| [Age_New=3] * rs3729856AA | .437 | .330 | 1.323 | .186 | -.210 | 1.085 |
| [Age_New=4] * rs3729856AA | -.041 | .040 | -1.019 | .308 | -.121 | .038 |
| [Age_New=5] * rs3729856AA | -.096 | .041 | -2.328 | .020 | -.177 | -.015 |
| [Age_New=1] * rs3729856G | .984 | .687 | 1.432 | .152 | -.363 | 2.331 |
| [Age_New=2] * rs3729856G | 1.023 | .496 | 2.062 | .039 | .050 | 1.995 |
| [Age_New=3] * rs3729856G | .448 | .332 | 1.348 | .178 | -.204 | 1.099 |
| [Age_New=4] * rs3729856G | 0^a^ |  |  |  |  |  |
| [Age_New=5] * rs3729856G | 0^a^ |  |  |  |  |  |
| [Age_New=1] * rs1062219C | .221 | .365 | .606 | .545 | -.494 | .936 |
| [Age_New=2] * rs1062219C | .018 | .033 | .542 | .588 | -.047 | .082 |
| [Age_New=3] * rs1062219C | -.018 | .033 | -.535 | .593 | -.083 | .047 |
| [Age_New=4] * rs1062219C | .038 | .489 | .078 | .938 | -.919 | .996 |
| [Age_New=5] * rs1062219C | .012 | .035 | .335 | .738 | -.056 | .080 |
| [Age_New=1] * rs1062219T | .261 | .365 | .716 | .474 | -.454 | .976 |
| [Age_New=2] * rs1062219T | 0^a^ |  |  |  |  |  |
| [Age_New=3] * rs1062219T | 0^a^ |  |  |  |  |  |
| [Age_New=4] * rs1062219T | -.038 | .489 | -.078 | .938 | -.996 | .919 |
| [Age_New=5] * rs1062219T | 0^a^ |  |  |  |  |  |
| [Age_New=1] * rs12825C | .204 | .268 | .760 | .447 | -.322 | .730 |
| [Age_New=2] * rs12825C | -.069 | .247 | -.280 | .780 | -.553 | .415 |
| [Age_New=3] * rs12825C | -.078 | .152 | -.515 | .607 | -.376 | .220 |
| [Age_New=4] * rs12825C | -.001 | .032 | -.034 | .973 | -.065 | .062 |
| [Age_New=5] * rs12825C | -.134 | .245 | -.546 | .585 | -.615 | .347 |
| [Age_New=1] * rs12825G | .165 | .267 | .616 | .538 | -.360 | .689 |
| [Age_New=2] * rs12825G | -.052 | .246 | -.210 | .833 | -.534 | .430 |
| [Age_New=3] * rs12825G | -.080 | .151 | -.530 | .596 | -.376 | .216 |
| [Age_New=4] * rs12825G | 0^a^ |  |  |  |  |  |
| [Age_New=5] * rs12825G | -.168 | .246 | -.683 | .495 | -.649 | .314 |
| [Age_New=1] * rs11785481C | .335 | .331 | 1.013 | .311 | -.313 | .983 |
| [Age_New=2] * rs11785481C | -.019 | .494 | -.039 | .969 | -.989 | .950 |
| [Age_New=3] * rs11785481C | .011 | .042 | .265 | .791 | -.071 | .093 |
| [Age_New=4] * rs11785481C | -.075 | .048 | -1.565 | .118 | -.168 | .019 |
| [Age_New=5] * rs11785481C | -.044 | .045 | -.978 | .328 | -.133 | .044 |
| [Age_New=1] * rs11785481T | .241 | .333 | .725 | .469 | -.411 | .894 |
| [Age_New=2] * rs11785481T | .004 | .497 | .007 | .994 | -.971 | .978 |
| [Age_New=3] * rs11785481T | 0^a^ |  |  |  |  |  |
| [Age_New=4] * rs11785481T | 0^a^ |  |  |  |  |  |
| [Age_New=5] * rs11785481T | 0^a^ |  |  |  |  |  |
| [Age_New=1] * rs3203358C | .323 | .460 | .703 | .482 | -.578 | 1.224 |
| [Age_New=2] * rs3203358C | -.192 | .672 | -.286 | .775 | -1.510 | 1.125 |
| [Age_New=3] * rs3203358C | -.024 | .035 | -.691 | .489 | -.092 | .044 |
| [Age_New=4] * rs3203358C | -.052 | .036 | -1.435 | .151 | -.122 | .019 |
| [Age_New=5] * rs3203358C | -.011 | .036 | -.308 | .758 | -.082 | .060 |
| [Age_New=1] * rs3203358G | .307 | .459 | .669 | .503 | -.593 | 1.207 |
| [Age_New=2] * rs3203358G | -.213 | .673 | -.316 | .752 | -1.533 | 1.107 |
| [Age_New=3] * rs3203358G | 0^a^ |  |  |  |  |  |
| [Age_New=4] * rs3203358G | 0^a^ |  |  |  |  |  |
| [Age_New=5] * rs3203358G | 0^a^ |  |  |  |  |  |
| [Age_New=1] * rs2740434G | -.331 | .441 | -.751 | .452 | -1.195 | .533 |
| [Age_New=2] * rs2740434G | .009 | .489 | .018 | .986 | -.949 | .966 |
| [Age_New=3] * rs2740434G | .477 | .260 | 1.838 | .066 | -.032 | .986 |
| [Age_New=4] * rs2740434G | .019 | .034 | .572 | .567 | -.047 | .086 |
| [Age_New=5] * rs2740434G | .033 | .495 | .067 | .947 | -.938 | 1.004 |
| [Age_New=1] * rs2740434AA | -.320 | .442 | -.723 | .469 | -1.186 | .547 |
| [Age_New=2] * rs2740434AA | .051 | .490 | .104 | .917 | -.909 | 1.011 |
| [Age_New=3] * rs2740434AA | .445 | .260 | 1.713 | .087 | -.064 | .955 |
| [Age_New=4] * rs2740434AA | 0^a^ |  |  |  |  |  |
| [Age_New=5] * rs2740434AA | .045 | .497 | .090 | .928 | -.929 | 1.018 |
| [Age_New=1] * rs17153743AA | .306 | .281 | 1.089 | .276 | -.245 | .857 |
| [Age_New=2] * rs17153743AA | -.048 | .092 | -.522 | .602 | -.227 | .132 |
| [Age_New=3] * rs17153743AA | -.045 | .358 | -.125 | .901 | -.746 | .656 |
| [Age_New=4] * rs17153743AA | .862 | .451 | 1.914 | .056 | -.021 | 1.745 |
| [Age_New=5] * rs17153743AA | .143 | .491 | .292 | .770 | -.819 | 1.105 |
| [Age_New=1] * rs17153743G | .169 | .302 | .559 | .576 | -.423 | .761 |
| [Age_New=2] * rs17153743G | 0^a^ |  |  |  |  |  |
| [Age_New=3] * rs17153743G | .238 | .375 | .634 | .526 | -.497 | .972 |
| [Age_New=4] * rs17153743G | .758 | .470 | 1.613 | .107 | -.163 | 1.678 |
| [Age_New=5] * rs17153743G | .136 | .503 | .271 | .787 | -.849 | 1.121 |
| [Age_New=1] * rs13264774C | .166 | .071 | 2.349 | .019 | .027 | .304 |
| [Age_New=2] * rs13264774C | .011 | .069 | .159 | .873 | -.124 | .146 |
| [Age_New=3] * rs13264774C | -.129 | .070 | -1.841 | .066 | -.267 | .008 |
| [Age_New=4] * rs13264774C | -.109 | .085 | -1.272 | .203 | -.276 | .059 |
| [Age_New=5] * rs13264774C | -.009 | .080 | -.114 | .909 | -.166 | .147 |
| [Age_New=1] * rs13264774T | .154 | .078 | 1.985 | .047 | .002 | .306 |
| [Age_New=2] * rs13264774T | -.081 | .077 | -1.057 | .291 | -.232 | .070 |
| [Age_New=3] * rs13264774T | -.157 | .078 | -2.022 | .043 | -.310 | -.005 |
| [Age_New=4] * rs13264774T | -.043 | .092 | -.467 | .641 | -.223 | .137 |
| [Age_New=5] * rs13264774T | -.038 | .087 | -.438 | .662 | -.209 | .132 |
| [Age_New=1] * rs804280AA | -.668 | .346 | -1.927 | .054 | -1.347 | .011 |
| [Age_New=2] * rs804280AA | -.546 | .347 | -1.575 | .115 | -1.225 | .133 |
| [Age_New=3] * rs804280AA | -.068 | .184 | -.370 | .711 | -.429 | .292 |
| [Age_New=4] * rs804280AA | .022 | .036 | .597 | .550 | -.049 | .093 |
| [Age_New=5] * rs804280AA | .375 | .248 | 1.514 | .130 | -.111 | .861 |
| [Age_New=1] * rs804280C | -.687 | .347 | -1.983 | .047 | -1.366 | -.008 |
| [Age_New=2] * rs804280C | -.508 | .347 | -1.465 | .143 | -1.188 | .172 |
| [Age_New=3] * rs804280C | -.070 | .183 | -.383 | .702 | -.428 | .288 |
| [Age_New=4] * rs804280C | 0^a^ |  |  |  |  |  |
| [Age_New=5] * rs804280C | .333 | .249 | 1.337 | .181 | -.155 | .820 |
| [Age_New=1] * rs804291AA | -.770 | .395 | -1.946 | .052 | -1.545 | .006 |
| [Age_New=2] * rs804291AA | -.018 | .037 | -.477 | .634 | -.090 | .055 |
| [Age_New=3] * rs804291AA | -.560 | .328 | -1.710 | .087 | -1.202 | .082 |
| [Age_New=4] * rs804291AA | .021 | .040 | .528 | .597 | -.058 | .100 |
| [Age_New=5] * rs804291AA | -.004 | .039 | -.093 | .926 | -.080 | .073 |
| [Age_New=1] * rs804291G | -.720 | .394 | -1.827 | .068 | -1.494 | .053 |
| [Age_New=2] * rs804291G | 0^a^ |  |  |  |  |  |
| [Age_New=3] * rs804291G | -.579 | .326 | -1.775 | .076 | -1.219 | .061 |
| [Age_New=4] * rs804291G | 0^a^ |  |  |  |  |  |
| [Age_New=5] * rs804291G | 0^a^ |  |  |  |  |  |

**HYPERCHOLESTEROLAEMIA** INTERACTION WITH AGE

| Parameter | B | Std. Error | t | Sig. | 95% Confidence Interval | |
| --- | --- | --- | --- | --- | --- | --- |
|  |  |  |  |  | Lower Bound | Upper Bound |
| Intercept | .897 | .284 | 3.159 | .002 | .340 | 1.453 |
| [Age_New=1] * rs3729855C | 1.044 | .400 | 2.611 | .009 | .260 | 1.828 |
| [Age_New=2] * rs3729855C | 1.487 | .738 | 2.016 | .044 | .041 | 2.934 |
| [Age_New=3] * rs3729855C | .277 | .363 | .762 | .446 | -.435 | .988 |
| [Age_New=4] * rs3729855C | -.470 | .355 | -1.322 | .186 | -1.166 | .227 |
| [Age_New=5] * rs3729855C | .280 | .453 | .619 | .536 | -.608 | 1.169 |
| [Age_New=1] * rs3729855T | 1.042 | .405 | 2.571 | .010 | .247 | 1.836 |
| [Age_New=2] * rs3729855T | 1.462 | .739 | 1.977 | .048 | .013 | 2.911 |
| [Age_New=3] * rs3729855T | .303 | .369 | .821 | .412 | -.420 | 1.025 |
| [Age_New=4] * rs3729855T | -.528 | .363 | -1.456 | .145 | -1.240 | .183 |
| [Age_New=5] * rs3729855T | .357 | .460 | .776 | .438 | -.545 | 1.260 |
| [Age_New=1] * rs3729856AA | -1.809 | .674 | -2.685 | .007 | -3.129 | -.488 |
| [Age_New=2] * rs3729856AA | .069 | .485 | .143 | .886 | -.882 | 1.021 |
| [Age_New=3] * rs3729856AA | .490 | .324 | 1.513 | .130 | -.145 | 1.126 |
| [Age_New=4] * rs3729856AA | -.012 | .039 | -.313 | .755 | -.089 | .065 |
| [Age_New=5] * rs3729856AA | .001 | .039 | .024 | .981 | -.076 | .078 |
| [Age_New=1] * rs3729856G | -1.786 | .674 | -2.652 | .008 | -3.106 | -.466 |
| [Age_New=2] * rs3729856G | -.003 | .486 | -.007 | .995 | -.956 | .950 |
| [Age_New=3] * rs3729856G | .519 | .326 | 1.592 | .111 | -.120 | 1.158 |
| [Age_New=4] * rs3729856G | 0^a^ |  |  |  |  |  |
| [Age_New=5] * rs3729856G | 0^a^ |  |  |  |  |  |
| [Age_New=1] * rs1062219C | -.570 | .358 | -1.592 | .111 | -1.271 | .132 |
| [Age_New=2] * rs1062219C | -.039 | .031 | -1.250 | .211 | -.101 | .022 |
| [Age_New=3] * rs1062219C | .013 | .032 | .408 | .683 | -.050 | .076 |
| [Age_New=4] * rs1062219C | .017 | .479 | .036 | .972 | -.922 | .956 |
| [Age_New=5] * rs1062219C | -.038 | .033 | -1.135 | .257 | -.103 | .028 |
| [Age_New=1] * rs1062219T | -.572 | .358 | -1.599 | .110 | -1.273 | .129 |
| [Age_New=2] * rs1062219T | 0^a^ |  |  |  |  |  |
| [Age_New=3] * rs1062219T | 0^a^ |  |  |  |  |  |
| [Age_New=4] * rs1062219T | -.017 | .479 | -.036 | .972 | -.956 | .922 |
| [Age_New=5] * rs1062219T | 0^a^ |  |  |  |  |  |
| [Age_New=1] * rs12825C | -.388 | .263 | -1.474 | .141 | -.904 | .128 |
| [Age_New=2] * rs12825C | -.506 | .242 | -2.090 | .037 | -.980 | -.031 |
| [Age_New=3] * rs12825C | -.210 | .164 | -1.278 | .201 | -.533 | .112 |
| [Age_New=4] * rs12825C | .024 | .031 | .772 | .440 | -.037 | .086 |
| [Age_New=5] * rs12825C | -.116 | .241 | -.482 | .630 | -.588 | .356 |
| [Age_New=1] * rs12825G | -.370 | .262 | -1.412 | .158 | -.884 | .144 |
| [Age_New=2] * rs12825G | -.523 | .241 | -2.169 | .030 | -.995 | -.050 |
| [Age_New=3] * rs12825G | -.198 | .164 | -1.211 | .226 | -.519 | .123 |
| [Age_New=4] * rs12825G | 0^a^ |  |  |  |  |  |
| [Age_New=5] * rs12825G | -.070 | .241 | -.290 | .772 | -.542 | .402 |
| [Age_New=1] * rs11785481C | -.419 | .325 | -1.290 | .197 | -1.055 | .217 |
| [Age_New=2] * rs11785481C | -.050 | .485 | -.103 | .918 | -1.000 | .900 |
| [Age_New=3] * rs11785481C | -.047 | .041 | -1.155 | .248 | -.126 | .033 |
| [Age_New=4] * rs11785481C | -.024 | .046 | -.528 | .597 | -.115 | .066 |
| [Age_New=5] * rs11785481C | -.102 | .043 | -2.349 | .019 | -.186 | -.017 |
| [Age_New=1] * rs11785481T | -.474 | .327 | -1.449 | .147 | -1.115 | .167 |
| [Age_New=2] * rs11785481T | -.064 | .487 | -.131 | .896 | -1.018 | .891 |
| [Age_New=3] * rs11785481T | 0^a^ |  |  |  |  |  |
| [Age_New=4] * rs11785481T | 0^a^ |  |  |  |  |  |
| [Age_New=5] * rs11785481T | 0^a^ |  |  |  |  |  |
| [Age_New=1] * rs3203358C | .884 | .451 | 1.960 | .050 | 2.025E-05 | 1.767 |
| [Age_New=2] * rs3203358C | -1.915 | .659 | -2.907 | .004 | -3.207 | -.624 |
| [Age_New=3] * rs3203358C | -.031 | .034 | -.921 | .357 | -.097 | .035 |
| [Age_New=4] * rs3203358C | .002 | .035 | .044 | .965 | -.066 | .069 |
| [Age_New=5] * rs3203358C | .017 | .035 | .498 | .618 | -.051 | .086 |
| [Age_New=1] * rs3203358G | .832 | .450 | 1.848 | .065 | -.050 | 1.714 |
| [Age_New=2] * rs3203358G | -1.989 | .660 | -3.014 | .003 | -3.283 | -.695 |
| [Age_New=3] * rs3203358G | 0^a^ |  |  |  |  |  |
| [Age_New=4] * rs3203358G | 0^a^ |  |  |  |  |  |
| [Age_New=5] * rs3203358G | 0^a^ |  |  |  |  |  |
| [Age_New=1] * rs2740434G | .288 | .432 | .667 | .505 | -.559 | 1.135 |
| [Age_New=2] * rs2740434G | 1.031 | .479 | 2.152 | .031 | .092 | 1.970 |
| [Age_New=3] * rs2740434G | .510 | .257 | 1.985 | .047 | .006 | 1.014 |
| [Age_New=4] * rs2740434G | .002 | .033 | .060 | .952 | -.062 | .066 |
| [Age_New=5] * rs2740434G | -1.056 | .485 | -2.177 | .029 | -2.007 | -.105 |
| [Age_New=1] * rs2740434AA | .282 | .434 | .652 | .515 | -.567 | 1.132 |
| [Age_New=2] * rs2740434AA | 1.082 | .480 | 2.253 | .024 | .141 | 2.024 |
| [Age_New=3] * rs2740434AA | .524 | .258 | 2.034 | .042 | .019 | 1.029 |
| [Age_New=4] * rs2740434AA | 0^a^ |  |  |  |  |  |
| [Age_New=5] * rs2740434AA | -1.038 | .486 | -2.134 | .033 | -1.992 | -.085 |
| [Age_New=1] * rs17153743AA | -.184 | .276 | -.666 | .505 | -.724 | .357 |
| [Age_New=2] * rs17153743AA | .007 | .088 | .075 | .940 | -.166 | .180 |
| [Age_New=3] * rs17153743AA | -.791 | .351 | -2.254 | .024 | -1.479 | -.103 |
| [Age_New=4] * rs17153743AA | .103 | .442 | .234 | .815 | -.763 | .969 |
| [Age_New=5] * rs17153743AA | .585 | .481 | 1.216 | .224 | -.358 | 1.528 |
| [Age_New=1] * rs17153743G | -.262 | .295 | -.887 | .375 | -.840 | .317 |
| [Age_New=2] * rs17153743G | 0^a^ |  |  |  |  |  |
| [Age_New=3] * rs17153743G | -1.046 | .368 | -2.840 | .005 | -1.768 | -.324 |
| [Age_New=4] * rs17153743G | -.102 | .458 | -.222 | .824 | -1.000 | .797 |
| [Age_New=5] * rs17153743G | .634 | .494 | 1.282 | .200 | -.335 | 1.603 |
| [Age_New=1] * rs13264774C | .060 | .072 | .833 | .405 | -.081 | .201 |
| [Age_New=2] * rs13264774C | .047 | .066 | .712 | .477 | -.083 | .177 |
| [Age_New=3] * rs13264774C | .006 | .068 | .081 | .935 | -.127 | .138 |
| [Age_New=4] * rs13264774C | -.096 | .086 | -1.115 | .265 | -.264 | .073 |
| [Age_New=5] * rs13264774C | -.075 | .075 | -1.000 | .317 | -.221 | .072 |
| [Age_New=1] * rs13264774T | .026 | .079 | .335 | .738 | -.128 | .180 |
| [Age_New=2] * rs13264774T | .053 | .074 | .719 | .472 | -.092 | .199 |
| [Age_New=3] * rs13264774T | .012 | .075 | .163 | .871 | -.135 | .160 |
| [Age_New=4] * rs13264774T | -.019 | .092 | -.208 | .836 | -.200 | .162 |
| [Age_New=5] * rs13264774T | -.096 | .082 | -1.174 | .240 | -.257 | .064 |
| [Age_New=1] * rs804280AA | .183 | .340 | .540 | .589 | -.482 | .849 |
| [Age_New=2] * rs804280AA | -.528 | .340 | -1.554 | .120 | -1.194 | .138 |
| [Age_New=3] * rs804280AA | -.138 | .181 | -.758 | .448 | -.493 | .218 |
| [Age_New=4] * rs804280AA | -.008 | .035 | -.236 | .813 | -.077 | .060 |
| [Age_New=5] * rs804280AA | -.028 | .243 | -.117 | .907 | -.504 | .447 |
| [Age_New=1] * rs804280C | .241 | .340 | .710 | .478 | -.425 | .907 |
| [Age_New=2] * rs804280C | -.539 | .340 | -1.586 | .113 | -1.205 | .127 |
| [Age_New=3] * rs804280C | -.125 | .180 | -.695 | .487 | -.478 | .228 |
| [Age_New=4] * rs804280C | 0^a^ |  |  |  |  |  |
| [Age_New=5] * rs804280C | -.071 | .244 | -.291 | .771 | -.549 | .407 |
| [Age_New=1] * rs804291AA | .221 | .388 | .570 | .568 | -.539 | .981 |
| [Age_New=2] * rs804291AA | -.062 | .035 | -1.754 | .079 | -.131 | .007 |
| [Age_New=3] * rs804291AA | -.548 | .322 | -1.706 | .088 | -1.179 | .082 |
| [Age_New=4] * rs804291AA | -.014 | .039 | -.366 | .715 | -.091 | .062 |
| [Age_New=5] * rs804291AA | .021 | .038 | .558 | .577 | -.053 | .095 |
| [Age_New=1] * rs804291G | .211 | .387 | .545 | .586 | -.547 | .969 |
| [Age_New=2] * rs804291G | 0^a^ |  |  |  |  |  |
| [Age_New=3] * rs804291G | -.567 | .320 | -1.769 | .077 | -1.195 | .061 |
| [Age_New=4] * rs804291G | 0^a^ |  |  |  |  |  |
| [Age_New=5] * rs804291G | 0^a^ |  |  |  |  |  |

**HYPERTRIGLYCERIDAEMIA** INTERACTION WITH AGE

| Parameter | B | Std. Error | t | Sig. | 95% Confidence Interval | |
| --- | --- | --- | --- | --- | --- | --- |
|  |  |  |  |  | Lower Bound | Upper Bound |
| Intercept | .191 | .262 | .729 | .466 | -.322 | .704 |
| [Age_New=1] * rs3729855C | 1.124 | .369 | 3.046 | .002 | .401 | 1.847 |
| [Age_New=2] * rs3729855C | -.257 | .714 | -.359 | .719 | -1.655 | 1.142 |
| [Age_New=3] * rs3729855C | .222 | .335 | .663 | .507 | -.434 | .878 |
| [Age_New=4] * rs3729855C | .255 | .328 | .779 | .436 | -.387 | .898 |
| [Age_New=5] * rs3729855C | -.072 | .418 | -.172 | .864 | -.891 | .748 |
| [Age_New=1] * rs3729855T | 1.157 | .374 | 3.095 | .002 | .424 | 1.890 |
| [Age_New=2] * rs3729855T | -.170 | .715 | -.238 | .812 | -1.572 | 1.232 |
| [Age_New=3] * rs3729855T | .317 | .340 | .932 | .352 | -.350 | .984 |
| [Age_New=4] * rs3729855T | .291 | .336 | .867 | .386 | -.367 | .949 |
| [Age_New=5] * rs3729855T | -.085 | .425 | -.200 | .841 | -.918 | .748 |
| [Age_New=1] * rs3729856AA | -2.139 | .621 | -3.442 | .001 | -3.357 | -.920 |
| [Age_New=2] * rs3729856AA | -.048 | .448 | -.107 | .915 | -.926 | .830 |
| [Age_New=3] * rs3729856AA | .640 | .299 | 2.142 | .032 | .054 | 1.227 |
| [Age_New=4] * rs3729856AA | .026 | .037 | .709 | .478 | -.046 | .098 |
| [Age_New=5] * rs3729856AA | .033 | .037 | .905 | .365 | -.039 | .106 |
| [Age_New=1] * rs3729856G | -2.139 | .621 | -3.443 | .001 | -3.357 | -.921 |
| [Age_New=2] * rs3729856G | -.080 | .448 | -.178 | .858 | -.959 | .799 |
| [Age_New=3] * rs3729856G | .675 | .301 | 2.245 | .025 | .086 | 1.264 |
| [Age_New=4] * rs3729856G | 0^a^ |  |  |  |  |  |
| [Age_New=5] * rs3729856G | 0^a^ |  |  |  |  |  |
| [Age_New=1] * rs1062219C | .026 | .330 | .078 | .938 | -.621 | .673 |
| [Age_New=2] * rs1062219C | .017 | .030 | .576 | .565 | -.041 | .075 |
| [Age_New=3] * rs1062219C | .022 | .030 | .736 | .462 | -.037 | .080 |
| [Age_New=4] * rs1062219C | -1.014 | .442 | -2.296 | .022 | -1.880 | -.148 |
| [Age_New=5] * rs1062219C | .075 | .031 | 2.416 | .016 | .014 | .136 |
| [Age_New=1] * rs1062219T | .036 | .330 | .108 | .914 | -.611 | .683 |
| [Age_New=2] * rs1062219T | 0^a^ |  |  |  |  |  |
| [Age_New=3] * rs1062219T | 0^a^ |  |  |  |  |  |
| [Age_New=4] * rs1062219T | -.986 | .442 | -2.231 | .026 | -1.852 | -.120 |
| [Age_New=5] * rs1062219T | 0^a^ |  |  |  |  |  |
| [Age_New=1] * rs12825C | -.304 | .243 | -1.252 | .211 | -.780 | .172 |
| [Age_New=2] * rs12825C | .363 | .316 | 1.147 | .251 | -.257 | .982 |
| [Age_New=3] * rs12825C | -.231 | .152 | -1.522 | .128 | -.528 | .066 |
| [Age_New=4] * rs12825C | 5.733E-05 | .029 | .002 | .998 | -.057 | .057 |
| [Age_New=5] * rs12825C | -.777 | .222 | -3.504 | .000 | -1.212 | -.342 |
| [Age_New=1] * rs12825G | -.345 | .242 | -1.428 | .153 | -.820 | .129 |
| [Age_New=2] * rs12825G | .361 | .315 | 1.145 | .252 | -.257 | .979 |
| [Age_New=3] * rs12825G | -.227 | .151 | -1.503 | .133 | -.523 | .069 |
| [Age_New=4] * rs12825G | 0^a^ |  |  |  |  |  |
| [Age_New=5] * rs12825G | -.821 | .222 | -3.696 | .000 | -1.257 | -.386 |
| [Age_New=1] * rs11785481C | .131 | .299 | .437 | .662 | -.456 | .718 |
| [Age_New=2] * rs11785481C | .039 | .447 | .088 | .930 | -.837 | .916 |
| [Age_New=3] * rs11785481C | .003 | .038 | .077 | .939 | -.071 | .077 |
| [Age_New=4] * rs11785481C | .048 | .043 | 1.113 | .266 | -.036 | .132 |
| [Age_New=5] * rs11785481C | -.077 | .040 | -1.900 | .057 | -.156 | .002 |
| [Age_New=1] * rs11785481T | .062 | .302 | .204 | .838 | -.530 | .653 |
| [Age_New=2] * rs11785481T | .025 | .449 | .055 | .956 | -.856 | .905 |
| [Age_New=3] * rs11785481T | 0^a^ |  |  |  |  |  |
| [Age_New=4] * rs11785481T | 0^a^ |  |  |  |  |  |
| [Age_New=5] * rs11785481T | 0^a^ |  |  |  |  |  |
| [Age_New=1] * rs3203358C | .853 | .416 | 2.051 | .040 | .038 | 1.668 |
| [Age_New=2] * rs3203358C | -.220 | .608 | -.362 | .717 | -1.411 | .971 |
| [Age_New=3] * rs3203358C | -.006 | .031 | -.185 | .853 | -.067 | .055 |
| [Age_New=4] * rs3203358C | -.049 | .032 | -1.510 | .131 | -.112 | .015 |
| [Age_New=5] * rs3203358C | -.082 | .033 | -2.495 | .013 | -.146 | -.017 |
| [Age_New=1] * rs3203358G | .780 | .415 | 1.880 | .060 | -.033 | 1.594 |
| [Age_New=2] * rs3203358G | -.226 | .609 | -.371 | .711 | -1.420 | .968 |
| [Age_New=3] * rs3203358G | 0^a^ |  |  |  |  |  |
| [Age_New=4] * rs3203358G | 0^a^ |  |  |  |  |  |
| [Age_New=5] * rs3203358G | 0^a^ |  |  |  |  |  |
| [Age_New=1] * rs2740434G | .068 | .399 | .171 | .865 | -.713 | .849 |
| [Age_New=2] * rs2740434G | 1.000 | .442 | 2.264 | .024 | .134 | 1.866 |
| [Age_New=3] * rs2740434G | -.210 | .237 | -.884 | .377 | -.674 | .255 |
| [Age_New=4] * rs2740434G | .023 | .031 | .743 | .458 | -.037 | .083 |
| [Age_New=5] * rs2740434G | -.070 | .447 | -.156 | .876 | -.947 | .808 |
| [Age_New=1] * rs2740434AA | .106 | .400 | .265 | .791 | -.678 | .890 |
| [Age_New=2] * rs2740434AA | 1.004 | .443 | 2.267 | .023 | .136 | 1.873 |
| [Age_New=3] * rs2740434AA | -.198 | .238 | -.835 | .404 | -.664 | .267 |
| [Age_New=4] * rs2740434AA | 0^a^ |  |  |  |  |  |
| [Age_New=5] * rs2740434AA | -.088 | .449 | -.197 | .844 | -.968 | .791 |
| [Age_New=1] * rs17153743AA | -.038 | .254 | -.150 | .881 | -.536 | .460 |
| [Age_New=2] * rs17153743AA | -.046 | .083 | -.557 | .577 | -.208 | .116 |
| [Age_New=3] * rs17153743AA | -.178 | .324 | -.548 | .583 | -.812 | .457 |
| [Age_New=4] * rs17153743AA | .809 | .407 | 1.986 | .047 | .010 | 1.608 |
| [Age_New=5] * rs17153743AA | 1.285 | .444 | 2.896 | .004 | .415 | 2.154 |
| [Age_New=1] * rs17153743G | -.053 | .272 | -.194 | .847 | -.586 | .481 |
| [Age_New=2] * rs17153743G | 0^a^ |  |  |  |  |  |
| [Age_New=3] * rs17153743G | -.152 | .341 | -.446 | .656 | -.819 | .516 |
| [Age_New=4] * rs17153743G | .640 | .423 | 1.514 | .130 | -.189 | 1.469 |
| [Age_New=5] * rs17153743G | 1.268 | .458 | 2.766 | .006 | .370 | 2.167 |
| [Age_New=1] * rs13264774C | .033 | .066 | .494 | .621 | -.097 | .163 |
| [Age_New=2] * rs13264774C | -.030 | .061 | -.482 | .630 | -.150 | .091 |
| [Age_New=3] * rs13264774C | .092 | .062 | 1.475 | .140 | -.030 | .215 |
| [Age_New=4] * rs13264774C | -.062 | .079 | -.787 | .431 | -.218 | .093 |
| [Age_New=5] * rs13264774C | -.015 | .069 | -.223 | .823 | -.150 | .120 |
| [Age_New=1] * rs13264774T | -.003 | .072 | -.036 | .971 | -.145 | .139 |
| [Age_New=2] * rs13264774T | -.034 | .069 | -.492 | .623 | -.168 | .101 |
| [Age_New=3] * rs13264774T | .132 | .069 | 1.895 | .058 | -.005 | .268 |
| [Age_New=4] * rs13264774T | .021 | .085 | .246 | .806 | -.146 | .188 |
| [Age_New=5] * rs13264774T | -.054 | .076 | -.709 | .478 | -.203 | .095 |
| [Age_New=1] * rs804280AA | .204 | .313 | .650 | .516 | -.411 | .818 |
| [Age_New=2] * rs804280AA | -.672 | .313 | -2.143 | .032 | -1.286 | -.057 |
| [Age_New=3] * rs804280AA | .050 | .167 | .301 | .764 | -.278 | .378 |
| [Age_New=4] * rs804280AA | -.003 | .032 | -.083 | .934 | -.066 | .061 |
| [Age_New=5] * rs804280AA | -.265 | .224 | -1.183 | .237 | -.704 | .174 |
| [Age_New=1] * rs804280C | .219 | .313 | .698 | .485 | -.395 | .833 |
| [Age_New=2] * rs804280C | -.634 | .313 | -2.022 | .043 | -1.248 | -.019 |
| [Age_New=3] * rs804280C | .095 | .166 | .570 | .569 | -.231 | .420 |
| [Age_New=4] * rs804280C | 0^a^ |  |  |  |  |  |
| [Age_New=5] * rs804280C | -.195 | .225 | -.867 | .386 | -.636 | .246 |
| [Age_New=1] * rs804291AA | .075 | .358 | .208 | .835 | -.627 | .776 |
| [Age_New=2] * rs804291AA | -.001 | .033 | -.018 | .986 | -.066 | .065 |
| [Age_New=3] * rs804291AA | -.302 | .297 | -1.018 | .309 | -.883 | .279 |
| [Age_New=4] * rs804291AA | .044 | .036 | 1.216 | .224 | -.027 | .116 |
| [Age_New=5] * rs804291AA | .012 | .035 | .351 | .725 | -.056 | .081 |
| [Age_New=1] * rs804291G | .138 | .357 | .387 | .699 | -.561 | .837 |
| [Age_New=2] * rs804291G | 0^a^ |  |  |  |  |  |
| [Age_New=3] * rs804291G | -.334 | .295 | -1.131 | .258 | -.913 | .245 |
| [Age_New=4] * rs804291G | 0^a^ |  |  |  |  |  |
| [Age_New=5] * rs804291G | 0^a^ |  |  |  |  |  |

**LOW HIGH DENSITY LIPOPROTEIN-CHOLESTERO** INTERACTION WITH AGE

| Parameter | B | Std. Error | t | Sig. | 95% Confidence Interval | |
| --- | --- | --- | --- | --- | --- | --- |
|  |  |  |  |  | Lower Bound | Upper Bound |
| Intercept | -.083 | .293 | -.282 | .778 | -.658 | .492 |
| [Age_New=1] * rs3729855C | .721 | .413 | 1.744 | .081 | -.089 | 1.531 |
| [Age_New=2] * rs3729855C | -.572 | .799 | -.716 | .474 | -2.139 | .995 |
| [Age_New=3] * rs3729855C | .241 | .375 | .641 | .521 | -.495 | .976 |
| [Age_New=4] * rs3729855C | .562 | .367 | 1.531 | .126 | -.158 | 1.283 |
| [Age_New=5] * rs3729855C | -.087 | .468 | -.185 | .853 | -1.005 | .831 |
| [Age_New=1] * rs3729855T | .610 | .419 | 1.456 | .145 | -.211 | 1.430 |
| [Age_New=2] * rs3729855T | -.386 | .801 | -.482 | .630 | -1.957 | 1.185 |
| [Age_New=3] * rs3729855T | .283 | .381 | .743 | .457 | -.464 | 1.030 |
| [Age_New=4] * rs3729855T | .691 | .376 | 1.837 | .066 | -.046 | 1.428 |
| [Age_New=5] * rs3729855T | -.160 | .476 | -.336 | .737 | -1.094 | .773 |
| [Age_New=1] * rs3729856AA | -.450 | .696 | -.646 | .518 | -1.814 | .915 |
| [Age_New=2] * rs3729856AA | .000 | .502 | .001 | .999 | -.983 | .984 |
| [Age_New=3] * rs3729856AA | -1.155 | .335 | -3.448 | .001 | -1.811 | -.498 |
| [Age_New=4] * rs3729856AA | -.056 | .041 | -1.351 | .177 | -.136 | .025 |
| [Age_New=5] * rs3729856AA | .026 | .041 | .640 | .522 | -.055 | .107 |
| [Age_New=1] * rs3729856G | -.443 | .696 | -.636 | .524 | -1.807 | .921 |
| [Age_New=2] * rs3729856G | -.009 | .502 | -.018 | .985 | -.994 | .976 |
| [Age_New=3] * rs3729856G | -1.136 | .337 | -3.371 | .001 | -1.796 | -.475 |
| [Age_New=4] * rs3729856G | 0^a^ |  |  |  |  |  |
| [Age_New=5] * rs3729856G | 0^a^ |  |  |  |  |  |
| [Age_New=1] * rs1062219C | .220 | .370 | .594 | .553 | -.505 | .944 |
| [Age_New=2] * rs1062219C | .025 | .033 | .747 | .455 | -.040 | .090 |
| [Age_New=3] * rs1062219C | .002 | .033 | .075 | .940 | -.063 | .068 |
| [Age_New=4] * rs1062219C | .034 | .495 | .070 | .945 | -.936 | 1.005 |
| [Age_New=5] * rs1062219C | .087 | .035 | 2.502 | .012 | .019 | .155 |
| [Age_New=1] * rs1062219T | .284 | .370 | .767 | .443 | -.441 | 1.008 |
| [Age_New=2] * rs1062219T | 0^a^ |  |  |  |  |  |
| [Age_New=3] * rs1062219T | 0^a^ |  |  |  |  |  |
| [Age_New=4] * rs1062219T | -.034 | .495 | -.070 | .945 | -1.005 | .936 |
| [Age_New=5] * rs1062219T | 0^a^ |  |  |  |  |  |
| [Age_New=1] * rs12825C | .122 | .272 | .449 | .654 | -.411 | .655 |
| [Age_New=2] * rs12825C | .394 | .354 | 1.113 | .266 | -.300 | 1.088 |
| [Age_New=3] * rs12825C | -.235 | .170 | -1.384 | .166 | -.568 | .098 |
| [Age_New=4] * rs12825C | .010 | .033 | .310 | .756 | -.054 | .074 |
| [Age_New=5] * rs12825C | -.049 | .249 | -.197 | .844 | -.536 | .438 |
| [Age_New=1] * rs12825G | .111 | .271 | .408 | .683 | -.421 | .642 |
| [Age_New=2] * rs12825G | .423 | .353 | 1.197 | .232 | -.270 | 1.115 |
| [Age_New=3] * rs12825G | -.252 | .169 | -1.492 | .136 | -.584 | .079 |
| [Age_New=4] * rs12825G | 0^a^ |  |  |  |  |  |
| [Age_New=5] * rs12825G | -.040 | .249 | -.161 | .872 | -.528 | .448 |
| [Age_New=1] * rs11785481C | .967 | .335 | 2.884 | .004 | .310 | 1.625 |
| [Age_New=2] * rs11785481C | -1.013 | .501 | -2.022 | .043 | -1.994 | -.031 |
| [Age_New=3] * rs11785481C | -.019 | .042 | -.440 | .660 | -.101 | .064 |
| [Age_New=4] * rs11785481C | .005 | .048 | .106 | .916 | -.089 | .100 |
| [Age_New=5] * rs11785481C | .017 | .045 | .369 | .712 | -.072 | .105 |
| [Age_New=1] * rs11785481T | .901 | .338 | 2.666 | .008 | .239 | 1.564 |
| [Age_New=2] * rs11785481T | -1.041 | .503 | -2.068 | .039 | -2.028 | -.054 |
| [Age_New=3] * rs11785481T | 0^a^ |  |  |  |  |  |
| [Age_New=4] * rs11785481T | 0^a^ |  |  |  |  |  |
| [Age_New=5] * rs11785481T | 0^a^ |  |  |  |  |  |
| [Age_New=1] * rs3203358C | -.772 | .466 | -1.659 | .097 | -1.685 | .140 |
| [Age_New=2] * rs3203358C | 1.171 | .681 | 1.720 | .085 | -.164 | 2.506 |
| [Age_New=3] * rs3203358C | .019 | .035 | .531 | .595 | -.050 | .087 |
| [Age_New=4] * rs3203358C | -.026 | .036 | -.708 | .479 | -.097 | .045 |
| [Age_New=5] * rs3203358C | -.016 | .037 | -.433 | .665 | -.088 | .056 |
| [Age_New=1] * rs3203358G | -.767 | .465 | -1.650 | .099 | -1.679 | .144 |
| [Age_New=2] * rs3203358G | 1.182 | .682 | 1.732 | .083 | -.155 | 2.519 |
| [Age_New=3] * rs3203358G | 0^a^ |  |  |  |  |  |
| [Age_New=4] * rs3203358G | 0^a^ |  |  |  |  |  |
| [Age_New=5] * rs3203358G | 0^a^ |  |  |  |  |  |
| [Age_New=1] * rs2740434G | -1.041 | .446 | -2.331 | .020 | -1.916 | -.165 |
| [Age_New=2] * rs2740434G | -.011 | .495 | -.022 | .982 | -.981 | .959 |
| [Age_New=3] * rs2740434G | .417 | .266 | 1.570 | .116 | -.104 | .937 |
| [Age_New=4] * rs2740434G | -.002 | .034 | -.059 | .953 | -.069 | .065 |
| [Age_New=5] * rs2740434G | 1.055 | .501 | 2.105 | .035 | .072 | 2.038 |
| [Age_New=1] * rs2740434AA | -1.072 | .448 | -2.392 | .017 | -1.950 | -.194 |
| [Age_New=2] * rs2740434AA | -.047 | .496 | -.095 | .924 | -1.020 | .926 |
| [Age_New=3] * rs2740434AA | .390 | .266 | 1.465 | .143 | -.132 | .912 |
| [Age_New=4] * rs2740434AA | 0^a^ |  |  |  |  |  |
| [Age_New=5] * rs2740434AA | 1.039 | .503 | 2.067 | .039 | .053 | 2.024 |
| [Age_New=1] * rs17153743AA | .601 | .285 | 2.111 | .035 | .043 | 1.159 |
| [Age_New=2] * rs17153743AA | .065 | .093 | .698 | .485 | -.117 | .246 |
| [Age_New=3] * rs17153743AA | 1.016 | .363 | 2.802 | .005 | .305 | 1.727 |
| [Age_New=4] * rs17153743AA | .083 | .456 | .182 | .856 | -.812 | .978 |
| [Age_New=5] * rs17153743AA | -.976 | .497 | -1.965 | .049 | -1.951 | -.002 |
| [Age_New=1] * rs17153743G | .624 | .305 | 2.049 | .041 | .027 | 1.222 |
| [Age_New=2] * rs17153743G | 0^a^ |  |  |  |  |  |
| [Age_New=3] * rs17153743G | 1.040 | .382 | 2.727 | .006 | .292 | 1.788 |
| [Age_New=4] * rs17153743G | .057 | .474 | .120 | .905 | -.872 | .985 |
| [Age_New=5] * rs17153743G | -1.171 | .514 | -2.280 | .023 | -2.178 | -.164 |
| [Age_New=1] * rs13264774C | -.012 | .074 | -.158 | .875 | -.158 | .134 |
| [Age_New=2] * rs13264774C | -.004 | .069 | -.053 | .958 | -.138 | .131 |
| [Age_New=3] * rs13264774C | .040 | .070 | .572 | .567 | -.097 | .177 |
| [Age_New=4] * rs13264774C | -.050 | .089 | -.564 | .573 | -.224 | .124 |
| [Age_New=5] * rs13264774C | .112 | .077 | 1.451 | .147 | -.039 | .263 |
| [Age_New=1] * rs13264774T | -.055 | .081 | -.676 | .499 | -.214 | .104 |
| [Age_New=2] * rs13264774T | -.039 | .077 | -.504 | .615 | -.190 | .112 |
| [Age_New=3] * rs13264774T | .059 | .078 | .764 | .445 | -.093 | .212 |
| [Age_New=4] * rs13264774T | -.015 | .095 | -.154 | .878 | -.202 | .172 |
| [Age_New=5] * rs13264774T | .078 | .085 | .916 | .360 | -.089 | .245 |
| [Age_New=1] * rs804280AA | .395 | .351 | 1.125 | .261 | -.293 | 1.083 |
| [Age_New=2] * rs804280AA | .482 | .351 | 1.374 | .169 | -.206 | 1.171 |
| [Age_New=3] * rs804280AA | .360 | .187 | 1.922 | .055 | -.007 | .728 |
| [Age_New=4] * rs804280AA | -.054 | .036 | -1.475 | .140 | -.125 | .018 |
| [Age_New=5] * rs804280AA | .385 | .251 | 1.533 | .125 | -.107 | .876 |
| [Age_New=1] * rs804280C | .348 | .351 | .991 | .322 | -.340 | 1.036 |
| [Age_New=2] * rs804280C | .513 | .351 | 1.461 | .144 | -.175 | 1.202 |
| [Age_New=3] * rs804280C | .412 | .186 | 2.216 | .027 | .048 | .777 |
| [Age_New=4] * rs804280C | 0^a^ |  |  |  |  |  |
| [Age_New=5] * rs804280C | .460 | .252 | 1.828 | .068 | -.033 | .954 |
| [Age_New=1] * rs804291AA | -.269 | .401 | -.672 | .502 | -1.055 | .516 |
| [Age_New=2] * rs804291AA | .022 | .037 | .589 | .556 | -.051 | .095 |
| [Age_New=3] * rs804291AA | -.113 | .332 | -.341 | .733 | -.764 | .538 |
| [Age_New=4] * rs804291AA | .031 | .041 | .749 | .454 | -.049 | .111 |
| [Age_New=5] * rs804291AA | -.018 | .039 | -.456 | .648 | -.095 | .059 |
| [Age_New=1] * rs804291G | -.264 | .400 | -.660 | .509 | -1.047 | .519 |
| [Age_New=2] * rs804291G | 0^a^ |  |  |  |  |  |
| [Age_New=3] * rs804291G | -.144 | .331 | -.435 | .664 | -.793 | .505 |
| [Age_New=4] * rs804291G | 0^a^ |  |  |  |  |  |
| [Age_New=5] * rs804291G | 0^a^ |  |  |  |  |  |

**HIGH LOW DENSITY LIPOPROTEIN-CHOLESTERO** INTERACTION WITH AGE

| Parameter | B | Std. Error | t | Sig. | 95% Confidence Interval | |
| --- | --- | --- | --- | --- | --- | --- |
|  |  |  |  |  | Lower Bound | Upper Bound |
| Intercept | .009 | .208 | .044 | .965 | -.399 | .417 |
| [Age_New=1] * rs3729855C | -.334 | .293 | -1.140 | .254 | -.909 | .241 |
| [Age_New=2] * rs3729855C | -.163 | .567 | -.288 | .774 | -1.276 | .949 |
| [Age_New=3] * rs3729855C | -.065 | .266 | -.243 | .808 | -.587 | .457 |
| [Age_New=4] * rs3729855C | .181 | .261 | .695 | .487 | -.330 | .692 |
| [Age_New=5] * rs3729855C | 1.139 | .332 | 3.426 | .001 | .487 | 1.790 |
| [Age_New=1] * rs3729855T | -.364 | .297 | -1.226 | .220 | -.947 | .218 |
| [Age_New=2] * rs3729855T | -.182 | .569 | -.320 | .749 | -1.297 | .933 |
| [Age_New=3] * rs3729855T | -.011 | .271 | -.042 | .966 | -.542 | .519 |
| [Age_New=4] * rs3729855T | .178 | .267 | .666 | .505 | -.345 | .701 |
| [Age_New=5] * rs3729855T | 1.162 | .338 | 3.439 | .001 | .500 | 1.825 |
| [Age_New=1] * rs3729856AA | .007 | .494 | .015 | .988 | -.961 | .976 |
| [Age_New=2] * rs3729856AA | .034 | .356 | .096 | .923 | -.664 | .732 |
| [Age_New=3] * rs3729856AA | .083 | .238 | .348 | .728 | -.383 | .549 |
| [Age_New=4] * rs3729856AA | -.024 | .029 | -.837 | .403 | -.082 | .033 |
| [Age_New=5] * rs3729856AA | -.021 | .029 | -.720 | .472 | -.079 | .036 |
| [Age_New=1] * rs3729856G | .028 | .494 | .056 | .955 | -.941 | .996 |
| [Age_New=2] * rs3729856G | -.025 | .357 | -.070 | .944 | -.724 | .674 |
| [Age_New=3] * rs3729856G | .092 | .239 | .385 | .701 | -.377 | .561 |
| [Age_New=4] * rs3729856G | 0^a^ |  |  |  |  |  |
| [Age_New=5] * rs3729856G | 0^a^ |  |  |  |  |  |
| [Age_New=1] * rs1062219C | .502 | .262 | 1.915 | .056 | -.012 | 1.017 |
| [Age_New=2] * rs1062219C | -.010 | .024 | -.406 | .685 | -.056 | .037 |
| [Age_New=3] * rs1062219C | .002 | .024 | .100 | .920 | -.044 | .049 |
| [Age_New=4] * rs1062219C | -.983 | .351 | -2.797 | .005 | -1.671 | -.294 |
| [Age_New=5] * rs1062219C | .012 | .025 | .505 | .613 | -.036 | .061 |
| [Age_New=1] * rs1062219T | .495 | .262 | 1.885 | .059 | -.020 | 1.009 |
| [Age_New=2] * rs1062219T | 0^a^ |  |  |  |  |  |
| [Age_New=3] * rs1062219T | 0^a^ |  |  |  |  |  |
| [Age_New=4] * rs1062219T | -1.017 | .351 | -2.896 | .004 | -1.706 | -.329 |
| [Age_New=5] * rs1062219T | 0^a^ |  |  |  |  |  |
| [Age_New=1] * rs12825C | -.204 | .193 | -1.055 | .291 | -.582 | .175 |
| [Age_New=2] * rs12825C | .230 | .251 | .914 | .361 | -.263 | .723 |
| [Age_New=3] * rs12825C | .123 | .121 | 1.022 | .307 | -.113 | .360 |
| [Age_New=4] * rs12825C | .009 | .023 | .398 | .690 | -.036 | .055 |
| [Age_New=5] * rs12825C | .120 | .176 | .680 | .496 | -.226 | .466 |
| [Age_New=1] * rs12825G | -.173 | .192 | -.902 | .367 | -.550 | .204 |
| [Age_New=2] * rs12825G | .231 | .251 | .923 | .356 | -.260 | .723 |
| [Age_New=3] * rs12825G | .153 | .120 | 1.275 | .202 | -.082 | .388 |
| [Age_New=4] * rs12825G | 0^a^ |  |  |  |  |  |
| [Age_New=5] * rs12825G | .115 | .177 | .652 | .514 | -.231 | .462 |
| [Age_New=1] * rs11785481C | -.010 | .238 | -.042 | .966 | -.477 | .457 |
| [Age_New=2] * rs11785481C | -.029 | .355 | -.083 | .934 | -.726 | .667 |
| [Age_New=3] * rs11785481C | -.036 | .030 | -1.216 | .224 | -.095 | .022 |
| [Age_New=4] * rs11785481C | -.010 | .034 | -.298 | .766 | -.077 | .057 |
| [Age_New=5] * rs11785481C | -.061 | .032 | -1.905 | .057 | -.124 | .002 |
| [Age_New=1] * rs11785481T | -.050 | .240 | -.209 | .834 | -.520 | .420 |
| [Age_New=2] * rs11785481T | -.009 | .357 | -.025 | .980 | -.709 | .691 |
| [Age_New=3] * rs11785481T | 0^a^ |  |  |  |  |  |
| [Age_New=4] * rs11785481T | 0^a^ |  |  |  |  |  |
| [Age_New=5] * rs11785481T | 0^a^ |  |  |  |  |  |
| [Age_New=1] * rs3203358C | -.036 | .331 | -.108 | .914 | -.684 | .612 |
| [Age_New=2] * rs3203358C | -.049 | .483 | -.100 | .920 | -.996 | .899 |
| [Age_New=3] * rs3203358C | -.012 | .025 | -.480 | .631 | -.061 | .037 |
| [Age_New=4] * rs3203358C | -.046 | .026 | -1.772 | .076 | -.096 | .005 |
| [Age_New=5] * rs3203358C | -.009 | .026 | -.341 | .733 | -.060 | .042 |
| [Age_New=1] * rs3203358G | -.057 | .330 | -.174 | .862 | -.704 | .589 |
| [Age_New=2] * rs3203358G | -.088 | .484 | -.182 | .855 | -1.037 | .861 |
| [Age_New=3] * rs3203358G | 0^a^ |  |  |  |  |  |
| [Age_New=4] * rs3203358G | 0^a^ |  |  |  |  |  |
| [Age_New=5] * rs3203358G | 0^a^ |  |  |  |  |  |
| [Age_New=1] * rs2740434G | .065 | .317 | .204 | .838 | -.557 | .686 |
| [Age_New=2] * rs2740434G | .018 | .351 | .051 | .959 | -.671 | .707 |
| [Age_New=3] * rs2740434G | .149 | .188 | .790 | .429 | -.221 | .518 |
| [Age_New=4] * rs2740434G | .009 | .024 | .388 | .698 | -.038 | .057 |
| [Age_New=5] * rs2740434G | -1.069 | .356 | -3.006 | .003 | -1.767 | -.372 |
| [Age_New=1] * rs2740434AA | .054 | .318 | .170 | .865 | -.569 | .677 |
| [Age_New=2] * rs2740434AA | .048 | .352 | .137 | .891 | -.642 | .739 |
| [Age_New=3] * rs2740434AA | .161 | .189 | .855 | .393 | -.209 | .532 |
| [Age_New=4] * rs2740434AA | 0^a^ |  |  |  |  |  |
| [Age_New=5] * rs2740434AA | -1.040 | .357 | -2.915 | .004 | -1.739 | -.341 |
| [Age_New=1] * rs17153743AA | -.461 | .202 | -2.282 | .023 | -.857 | -.065 |
| [Age_New=2] * rs17153743AA | -.052 | .067 | -.775 | .438 | -.183 | .079 |
| [Age_New=3] * rs17153743AA | .010 | .257 | .038 | .969 | -.495 | .514 |
| [Age_New=4] * rs17153743AA | .991 | .324 | 3.058 | .002 | .356 | 1.626 |
| [Age_New=5] * rs17153743AA | -.095 | .353 | -.268 | .789 | -.786 | .597 |
| [Age_New=1] * rs17153743G | -.528 | .216 | -2.442 | .015 | -.952 | -.104 |
| [Age_New=2] * rs17153743G | 0^a^ |  |  |  |  |  |
| [Age_New=3] * rs17153743G | .014 | .271 | .052 | .958 | -.518 | .546 |
| [Age_New=4] * rs17153743G | 1.010 | .336 | 3.005 | .003 | .351 | 1.669 |
| [Age_New=5] * rs17153743G | -.073 | .365 | -.201 | .841 | -.788 | .641 |
| [Age_New=1] * rs13264774C | -.011 | .053 | -.214 | .831 | -.115 | .092 |
| [Age_New=2] * rs13264774C | .023 | .049 | .471 | .638 | -.073 | .118 |
| [Age_New=3] * rs13264774C | -.087 | .051 | -1.724 | .085 | -.186 | .012 |
| [Age_New=4] * rs13264774C | .031 | .063 | .494 | .621 | -.092 | .155 |
| [Age_New=5] * rs13264774C | -.074 | .055 | -1.348 | .178 | -.181 | .034 |
| [Age_New=1] * rs13264774T | -.052 | .058 | -.904 | .366 | -.165 | .061 |
| [Age_New=2] * rs13264774T | .014 | .055 | .259 | .796 | -.093 | .121 |
| [Age_New=3] * rs13264774T | -.079 | .056 | -1.416 | .157 | -.189 | .030 |
| [Age_New=4] * rs13264774T | .047 | .068 | .691 | .490 | -.086 | .180 |
| [Age_New=5] * rs13264774T | -.064 | .060 | -1.059 | .290 | -.183 | .055 |
| [Age_New=1] * rs804280AA | .110 | .249 | .442 | .658 | -.378 | .599 |
| [Age_New=2] * rs804280AA | .185 | .249 | .741 | .458 | -.304 | .673 |
| [Age_New=3] * rs804280AA | -.118 | .133 | -.886 | .375 | -.379 | .143 |
| [Age_New=4] * rs804280AA | -.006 | .026 | -.227 | .820 | -.056 | .045 |
| [Age_New=5] * rs804280AA | .172 | .178 | .966 | .334 | -.177 | .521 |
| [Age_New=1] * rs804280C | .145 | .249 | .580 | .562 | -.344 | .633 |
| [Age_New=2] * rs804280C | .179 | .249 | .717 | .474 | -.310 | .667 |
| [Age_New=3] * rs804280C | -.123 | .132 | -.931 | .352 | -.382 | .136 |
| [Age_New=4] * rs804280C | 0^a^ |  |  |  |  |  |
| [Age_New=5] * rs804280C | .142 | .179 | .797 | .425 | -.208 | .493 |
| [Age_New=1] * rs804291AA | .486 | .284 | 1.711 | .087 | -.071 | 1.044 |
| [Age_New=2] * rs804291AA | -.036 | .027 | -1.349 | .177 | -.088 | .016 |
| [Age_New=3] * rs804291AA | .057 | .236 | .244 | .807 | -.405 | .520 |
| [Age_New=4] * rs804291AA | .005 | .029 | .166 | .868 | -.052 | .062 |
| [Age_New=5] * rs804291AA | -.016 | .028 | -.572 | .567 | -.071 | .039 |
| [Age_New=1] * rs804291G | .476 | .284 | 1.679 | .093 | -.080 | 1.032 |
| [Age_New=2] * rs804291G | 0^a^ |  |  |  |  |  |
| [Age_New=3] * rs804291G | .040 | .235 | .171 | .864 | -.420 | .501 |
| [Age_New=4] * rs804291G | 0^a^ |  |  |  |  |  |
| [Age_New=5] * rs804291G | 0^a^ |  |  |  |  |  |

**MYOCARDIAL INFARCTION** INTERACTION WITH AGE

| Parameter | B | Std. Error | t | P-value | Bonferroni | Adjusted P-value | 95% Confidence Interval | |
| --- | --- | --- | --- | --- | --- | --- | --- | --- |
|  |  |  |  |  |  |  | Lower Bound | Upper Bound |
| Intercept | .531 | .254 | 2.088 | .037 |  |  | .032 | 1.029 |
| [Age_New=2] * rs1062219C | .059 | .027 | 2.145 | .032 | .128 | FALSE | .005 | .113 |
| [Age_New=2] * rs13264774C | .172 | .058 | 2.963 | .003 | .012 | TRUE | .058 | .285 |
| [Age_New=2] * rs13264774T | .173 | .065 | 2.674 | .008 | .030 | TRUE | .046 | .301 |
| [Age_New=1] * rs804280AA | -.685 | .304 | -2.254 | .024 | .097 | FALSE | -1.282 | -.089 |
| [Age_New=3] * rs804280AA | .362 | .161 | 2.253 | .024 | .097 | FALSE | .047 | .677 |
| [Age_New=1] * rs804280C | -.676 | .304 | -2.222 | .026 | .105 | FALSE | -1.272 | -.080 |
| [Age_New=3] * rs804280C | .425 | .160 | 2.663 | .008 | .031 | TRUE | .112 | .738 |

**AGE LEGEND:**

1. Age<=44
2. 44<Age<=53
3. 53<Age<=61
4. 61<Age<=68
5. Age>68

Significant After Bonferroni Correction

Significant Before Bonferroni Correction (P<=0.05)

**TYPE 2 DIABETES MELLITUS** INTERACTION WITH AGE

| Parameter | B | Std. Error | t | P-value | Bonferroni | Adjusted  P-value | 95% Confidence Interval | |
| --- | --- | --- | --- | --- | --- | --- | --- | --- |
|  |  |  |  |  |  |  | Lower Bound | Upper Bound |
| Intercept | .655 | .275 | 2.382 | .017 | .069 | FALSE | .116 | 1.194 |
| [Age_New=4] * rs3729855C | .759 | .343 | 2.214 | .027 | .107 | FALSE | .087 | 1.430 |
| [Age_New=3] * rs3729856AA | .745 | .313 | 2.376 | .018 | .070 | FALSE | .130 | 1.359 |
| [Age_New=3] * rs3729856G | .756 | .315 | 2.398 | .016 | .066 | FALSE | .138 | 1.374 |
| [Age_New=3] * rs12825C | .505 | .144 | 3.501 | .000 | .002 | TRUE | .222 | .788 |
| [Age_New=3] * rs12825G | .480 | .143 | 3.349 | .001 | .003 | TRUE | .199 | .761 |
| [Age_New=2] * rs11785481C | .966 | .469 | 2.059 | .040 | .158 | FALSE | .046 | 1.886 |
| [Age_New=2] * rs11785481T | 1.055 | .471 | 2.238 | .025 | .101 | FALSE | .131 | 1.979 |
| [Age_New=5] * rs2740434AA | -.934 | .471 | -1.982 | .047 | .190 | FALSE | -1.858 | -.010 |
| [Age_New=3] * rs13264774C | .168 | .063 | 2.657 | .008 | .032 | TRUE | .044 | .292 |
| [Age_New=3] * rs13264774T | .151 | .070 | 2.145 | .032 | .128 | FALSE | .013 | .289 |
| [Age_New=1] * rs804280AA | -.792 | .329 | -2.407 | .016 | .064 | FALSE | -1.437 | -.147 |
| [Age_New=3] * rs804280AA | -.448 | .174 | -2.576 | .010 | .040 | TRUE | -.789 | -.107 |
| [Age_New=5] * rs804280AA | .620 | .235 | 2.636 | .008 | .034 | TRUE | .159 | 1.081 |
| [Age_New=1] * rs804280C | -.799 | .329 | -2.429 | .015 | .061 | FALSE | -1.445 | -.154 |
| [Age_New=3] * rs804280C | -.371 | .173 | -2.147 | .032 | .127 | FALSE | -.710 | -.032 |
| [Age_New=5] * rs804280C | .647 | .236 | 2.741 | .006 | .025 | TRUE | .184 | 1.109 |

**HYPERTENSION** INTERACTION WITH AGE

| Parameter | B | Std. Error | t | Sig. | Bonferroni | Adjusted P-values | 95% Confidence Interval | |
| --- | --- | --- | --- | --- | --- | --- | --- | --- |
|  |  |  |  |  |  |  | Lower Bound | Upper Bound |
| [Age_New=1] * rs12825C | -.793 | .216 | -3.670 | .000 | .001 | TRUE | -1.216 | -.369 |
| [Age_New=1] * rs12825G | -.809 | .215 | -3.760 | .000 | .001 | TRUE | -1.231 | -.387 |
| [Age_New=5] * rs12825G | .395 | .198 | 1.999 | .046 | .183 | FALSE | .008 | .783 |
| [Age_New=1] * rs11785481C | -.703 | .266 | -2.641 | .008 | .033 | TRUE | -1.224 | -.181 |
| [Age_New=4] * rs11785481C | -.086 | .038 | -2.297 | .022 | .087 | FALSE | -.160 | -.013 |
| [Age_New=1] * rs11785481T | -.734 | .268 | -2.740 | .006 | .025 | TRUE | -1.259 | -.209 |
| [Age_New=3] * rs2740434G | .550 | .209 | 2.632 | .008 | .034 | TRUE | .140 | .959 |
| [Age_New=4] * rs2740434G | -.053 | .026 | -2.020 | .043 | .174 | FALSE | -.105 | -.002 |
| [Age_New=3] * rs2740434AA | .559 | .209 | 2.675 | .007 | .030 | TRUE | .149 | .969 |
| [Age_New=1] * rs17153743AA | -.522 | .226 | -2.310 | .021 | .084 | FALSE | -.965 | -.079 |
| [Age_New=2] * rs17153743AA | -.196 | .071 | -2.750 | .006 | .024 | TRUE | -.336 | -.056 |
| [Age_New=5] * rs17153743AA | -.942 | .395 | -2.387 | .017 | .068 | FALSE | -1.716 | -.168 |
| [Age_New=5] * rs17153743G | -.873 | .403 | -2.164 | .031 | .122 | FALSE | -1.664 | -.082 |
| [Age_New=1] * rs804291AA | 1.101 | .318 | 3.460 | .001 | .002 | TRUE | .477 | 1.725 |
| [Age_New=3] * rs804291AA | -.727 | .263 | -2.761 | .006 | .023 | TRUE | -1.244 | -.211 |
| [Age_New=1] * rs804291G | 1.132 | .317 | 3.567 | .000 | .001 | TRUE | .510 | 1.754 |
| [Age_New=3] * rs804291G | -.734 | .263 | -2.797 | .005 | .021 | TRUE | -1.249 | -.220 |

**OBESITY** INTERACTION WITH AGE

| **Parameter** | **B** | **Std. Error** | **t** | **Sig.** | **Bonferroni** | **P-Value** | **95% Confidence Interval** | |
| --- | --- | --- | --- | --- | --- | --- | --- | --- |
|  |  |  |  |  |  |  | **Lower Bound** | **Upper Bound** |
| [Age_New=2] * rs3729856AA | 1.011 | .495 | 2.040 | .041 | 0.1657 | FALSE | .039 | 1.982 |
| [Age_New=5] * rs3729856AA | -.096 | .041 | -2.328 | .020 | 0.0797 | FALSE | -.177 | -.015 |
| [Age_New=2] * rs3729856G | 1.023 | .496 | 2.062 | .039 | 0.1570 | FALSE | .050 | 1.995 |
| [Age_New=1] * rs13264774C | .166 | .071 | 2.349 | .019 | 0.0755 | FALSE | .027 | .304 |
| [Age_New=1] * rs13264774T | .154 | .078 | 1.985 | .047 | 0.1888 | FALSE | .002 | .306 |
| [Age_New=3] * rs13264774T | -.157 | .078 | -2.022 | .043 | 0.1729 | FALSE | -.310 | -.005 |
| [Age_New=1] * rs804280C | -.687 | .347 | -1.983 | .047 | 0.1898 | FALSE | -1.366 | -.008 |

**Hypercholesterolaemia Interaction with age**

| **Parameter** | **B** | **Std. Error** | **t** | **Sig.** | **Bonferroni** | **P-Value** | **95% Confidence Interval** | |
| --- | --- | --- | --- | --- | --- | --- | --- | --- |
|  |  |  |  |  |  |  | **Lower Bound** | **Upper Bound** |
| Intercept | .897 | .284 | 3.159 | .002 |  |  | .340 | 1.453 |
| [Age_New=1] * rs3729855C | 1.044 | .400 | 2.611 | .009 | .036 | TRUE | .260 | 1.828 |
| [Age_New=2] * rs3729855C | 1.487 | .738 | 2.016 | .044 | .175 | FALSE | .041 | 2.934 |
| [Age_New=1] * rs3729855T | 1.042 | .405 | 2.571 | .010 | .041 | TRUE | .247 | 1.836 |
| [Age_New=2] * rs3729855T | 1.462 | .739 | 1.977 | .048 | .192 | FALSE | .013 | 2.911 |
| [Age_New=1] * rs3729856AA | -1.809 | .674 | -2.685 | .007 | .029 | TRUE | -3.129 | -.488 |
| [Age_New=1] * rs3729856G | -1.786 | .674 | -2.652 | .008 | .032 | TRUE | -3.106 | -.466 |
| [Age_New=2] * rs12825C | -.506 | .242 | -2.090 | .037 | .147 | FALSE | -.980 | -.031 |
| [Age_New=2] * rs12825G | -.523 | .241 | -2.169 | .030 | .120 | FALSE | -.995 | -.050 |
| [Age_New=5] * rs11785481C | -.102 | .043 | -2.349 | .019 | .075 | FALSE | -.186 | -.017 |
| [Age_New=1] * rs3203358C | .884 | .451 | 1.960 | .050 | .200 | FALSE | 2.025E-05 | 1.767 |
| [Age_New=2] * rs3203358C | -1.915 | .659 | -2.907 | .004 | .015 | TRUE | -3.207 | -.624 |
| [Age_New=2] * rs3203358G | -1.989 | .660 | -3.014 | .003 | .010 | TRUE | -3.283 | -.695 |
| [Age_New=2] * rs2740434G | 1.031 | .479 | 2.152 | .031 | .126 | FALSE | .092 | 1.970 |
| [Age_New=3] * rs2740434G | .510 | .257 | 1.985 | .047 | .189 | FALSE | .006 | 1.014 |
| [Age_New=5] * rs2740434G | -1.056 | .485 | -2.177 | .029 | .118 | FALSE | -2.007 | -.105 |
| [Age_New=2] * rs2740434AA | 1.082 | .480 | 2.253 | .024 | .097 | FALSE | .141 | 2.024 |
| [Age_New=3] * rs2740434AA | .524 | .258 | 2.034 | .042 | .168 | FALSE | .019 | 1.029 |
| [Age_New=5] * rs2740434AA | -1.038 | .486 | -2.134 | .033 | .131 | FALSE | -1.992 | -.085 |
| [Age_New=3] * rs17153743AA | -.791 | .351 | -2.254 | .024 | .097 | FALSE | -1.479 | -.103 |
| [Age_New=3] * rs17153743G | -1.046 | .368 | -2.840 | .005 | .018 | TRUE | -1.768 | -.324 |

**HYPERTRIGLYCERIDAEMIA** INTERACTION WITH AGE

| **Parameter** | **B** | **Std. Error** | **t** | **Sig.** | **Bonferroni** | **P-Value** | **95% Confidence Interval** | |
| --- | --- | --- | --- | --- | --- | --- | --- | --- |
|  |  |  |  |  |  |  | **Lower Bound** | **Upper Bound** |
| [Age_New=1] * rs3729855C | 1.124 | .369 | 3.046 | .002 | .009 | TRUE | .401 | 1.847 |
| [Age_New=1] * rs3729855T | 1.157 | .374 | 3.095 | .002 | .008 | TRUE | .424 | 1.890 |
| [Age_New=1] * rs3729856AA | -2.139 | .621 | -3.442 | .001 | .002 | TRUE | -3.357 | -.920 |
| [Age_New=3] * rs3729856AA | .640 | .299 | 2.142 | .032 | .129 | FALSE | .054 | 1.227 |
| [Age_New=1] * rs3729856G | -2.139 | .621 | -3.443 | .001 | .002 | TRUE | -3.357 | -.921 |
| [Age_New=3] * rs3729856G | .675 | .301 | 2.245 | .025 | .099 | FALSE | .086 | 1.264 |
| [Age_New=4] * rs1062219C | -1.014 | .442 | -2.296 | .022 | .087 | FALSE | -1.880 | -.148 |
| [Age_New=5] * rs1062219C | .075 | .031 | 2.416 | .016 | .063 | FALSE | .014 | .136 |
| [Age_New=4] * rs1062219T | -.986 | .442 | -2.231 | .026 | .103 | FALSE | -1.852 | -.120 |
| [Age_New=5] * rs12825C | -.777 | .222 | -3.504 | .000 | .002 | TRUE | -1.212 | -.342 |
| [Age_New=5] * rs12825G | -.821 | .222 | -3.696 | .000 | .001 | TRUE | -1.257 | -.386 |
| [Age_New=1] * rs3203358C | .853 | .416 | 2.051 | .040 | .161 | FALSE | .038 | 1.668 |
| [Age_New=5] * rs3203358C | -.082 | .033 | -2.495 | .013 | .050 | FALSE | -.146 | -.017 |
| [Age_New=2] * rs2740434G | 1.000 | .442 | 2.264 | .024 | .094 | FALSE | .134 | 1.866 |
| [Age_New=2] * rs2740434AA | 1.004 | .443 | 2.267 | .023 | .094 | FALSE | .136 | 1.873 |
| [Age_New=4] * rs17153743AA | .809 | .407 | 1.986 | .047 | .188 | FALSE | .010 | 1.608 |
| [Age_New=5] * rs17153743AA | 1.285 | .444 | 2.896 | .004 | .015 | TRUE | .415 | 2.154 |
| [Age_New=5] * rs17153743G | 1.268 | .458 | 2.766 | .006 | .023 | TRUE | .370 | 2.167 |
| [Age_New=2] * rs804280AA | -.672 | .313 | -2.143 | .032 | .128 | FALSE | -1.286 | -.057 |
| [Age_New=2] * rs804280C | -.634 | .313 | -2.022 | .043 | .173 | FALSE | -1.248 | -.019 |

**LOW HIGH DENSITY LIPOPROTEIN-CHOLESTERO** INTERACTION WITH AGE

| **Parameter** | **B** | **Std. Error** | **t** | **Sig.** | **Bonferroni** | **P-Value** | **95% Confidence Interval** | |
| --- | --- | --- | --- | --- | --- | --- | --- | --- |
|  |  |  |  |  |  |  | **Lower Bound** | **Upper Bound** |
| [Age_New=3] * rs3729856AA | -1.155 | .335 | -3.448 | .001 | 0.002 | TRUE | -1.811 | -.498 |
| [Age_New=3] * rs3729856G | -1.136 | .337 | -3.371 | .001 | 0.003 | TRUE | -1.796 | -.475 |
| [Age_New=5] * rs1062219C | .087 | .035 | 2.502 | .012 | .049 | TRUE | .019 | .155 |
| [Age_New=1] * rs11785481C | .967 | .335 | 2.884 | .004 | .016 | TRUE | .310 | 1.625 |
| [Age_New=2] * rs11785481C | -1.013 | .501 | -2.022 | .043 | 0.173 | FALSE | -1.994 | -.031 |
| [Age_New=1] * rs11785481T | .901 | .338 | 2.666 | .008 | .031 | TRUE | .239 | 1.564 |
| [Age_New=2] * rs11785481T | -1.041 | .503 | -2.068 | .039 | 0.155 | FALSE | -2.028 | -.054 |
| [Age_New=1] * rs2740434G | -1.041 | .446 | -2.331 | .020 | 0.079 | FALSE | -1.916 | -.165 |
| [Age_New=5] * rs2740434G | 1.055 | .501 | 2.105 | .035 | .141 | FALSE | .072 | 2.038 |
| [Age_New=1] * rs2740434AA | -1.072 | .448 | -2.392 | .017 | 0.067 | FALSE | -1.950 | -.194 |
| [Age_New=5] * rs2740434AA | 1.039 | .503 | 2.067 | .039 | .155 | FALSE | .053 | 2.024 |
| [Age_New=1] * rs17153743AA | .601 | .285 | 2.111 | .035 | .139 | FALSE | .043 | 1.159 |
| [Age_New=3] * rs17153743AA | 1.016 | .363 | 2.802 | .005 | .020 | TRUE | .305 | 1.727 |
| [Age_New=5] * rs17153743AA | -.976 | .497 | -1.965 | .049 | 0.198 | FALSE | -1.951 | -.002 |
| [Age_New=1] * rs17153743G | .624 | .305 | 2.049 | .041 | .162 | FALSE | .027 | 1.222 |
| [Age_New=3] * rs17153743G | 1.040 | .382 | 2.727 | .006 | .026 | TRUE | .292 | 1.788 |
| [Age_New=5] * rs17153743G | -1.171 | .514 | -2.280 | .023 | 0.091 | FALSE | -2.178 | -.164 |
| [Age_New=3] * rs804280C | .412 | .186 | 2.216 | .027 | .107 | FALSE | .048 | .777 |

**HIGH LOW DENSITY LIPOPROTEIN-CHOLESTERO** INTERACTION WITH AGE

| **Parameter** | **B** | **Std. Error** | **t** | **Sig.** | **Bonferroni** | **P-Value** | **95% Confidence Interval** | |
| --- | --- | --- | --- | --- | --- | --- | --- | --- |
|  |  |  |  |  |  |  | **Lower Bound** | **Upper Bound** |
| [Age_New=5] * rs3729855C | 1.139 | .332 | 3.426 | .001 | .002 | TRUE | .487 | 1.790 |
| [Age_New=5] * rs3729855T | 1.162 | .338 | 3.439 | .001 | .002 | TRUE | .500 | 1.825 |
| [Age_New=4] * rs1062219C | -.983 | .351 | -2.797 | .005 | 0.021 | TRUE | -1.671 | -.294 |
| [Age_New=4] * rs1062219T | -1.017 | .351 | -2.896 | .004 | 0.015 | TRUE | -1.706 | -.329 |
| [Age_New=5] * rs2740434G | -1.069 | .356 | -3.006 | .003 | 0.011 | TRUE | -1.767 | -.372 |
| [Age_New=5] * rs2740434AA | -1.040 | .357 | -2.915 | .004 | 0.014 | TRUE | -1.739 | -.341 |
| [Age_New=1] * rs17153743AA | -.461 | .202 | -2.282 | .023 | .090 | FALSE | -.857 | -.065 |
| [Age_New=4] * rs17153743AA | .991 | .324 | 3.058 | .002 | .009 | TRUE | .356 | 1.626 |
| [Age_New=1] * rs17153743G | -.528 | .216 | -2.442 | .015 | .059 | FALSE | -.952 | -.104 |
| [Age_New=4] * rs17153743G | 1.010 | .336 | 3.005 | .003 | .011 | TRUE | .351 | 1.669 |
